# Light-gated electro(de)wetting with photoswitchable amphiphiles

**DOI:** 10.1140/epje/s10189-026-00632-5

**Published:** 2026-09-14

**Authors:** Billura Shakhayeva, Michael Hardt, Björn Braunschweig

**Affiliations:** Institute of Physical Chemistry and Center for Soft Nanoscience, University of Münster, Corrensstraße 28/30, 48149 Münster, Germany

## Abstract

We present an approach for controlling the surface wettability with two triggers through a combination of electrodewetting (EDeW) and photoswitchable arylazopyrazole-triethylammonium bromide surfactants (AAP-TB). Experiments were performed on hydrophilic Si/SiO_2_ substrates using surfactant-laden aqueous droplets with a solution pH of 2.2. Two different cationic photoswitches were studied which differed in their terminal alkyl group. Here, either hydrogenated C_0_AAP-TB or butyl-terminated C_4_AAP-TB were applied. Pendant drop tensiometry revealed pronounced, light-dependent differences in surface tension and in the surfactants’ critical micelle concentrations (CMC) through *E*/*Z* photoisomerization. The more surface-active C_4_AAP-TB, exhibited the largest changes in surface tension upon *E*/*Z* isomerization of up to ~20 mN m^-1^. Applying potentials of +3 V for C_0_AAP-TB and +4 V for C_4_AAP-TB induces dewetting of surfactant-laden aqueous drops on the substrate surfaces. The change in contact angle $\Delta \theta$ after application of a potential was strongly dependent on surfactant concentration, where a maximum in $\Delta \theta$ of 13° and 7° for C_0_AAP-TB and for C_4_AAP-TB surfactants, respectively, was observed at about 1/10 of the respective CMCs of the surfactants’ E isomer. In addition, in situ light irradiation superimposed to the driving potential for EDeW triggers *E*/*Z* photoisomerization and modulates the contact angle reversibly by several degrees and with small hysteresis. Furthermore, at specific concentrations, the interplay of electric fields and spatially non-uniform photoisomerization caused by irradiation with intense UV light generates surface tension gradients that resulted in droplet deformation, droplet splitting, and ultimately in a directed motion of drops. Thus, combining potential-induced EDeW and surface energy changes by *E*/*Z* isomerization of AAP-TB surfactants provides reversible control over wettability and offers a versatile platform for programmable manipulation of liquid interfaces.

**Graphic abstract:**

**Figure:**
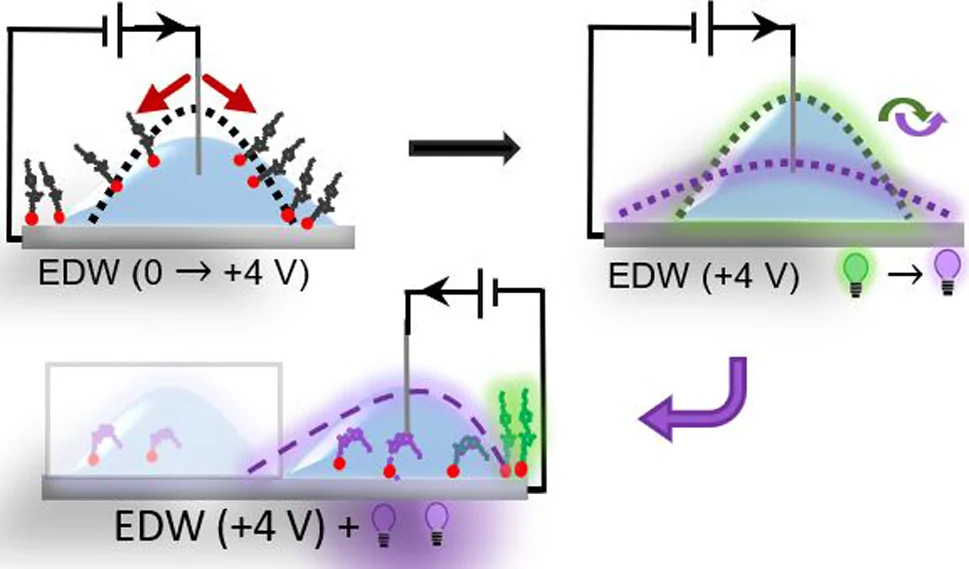
Changes in apparent contact angle and drop splitting due to a combination of light irradiation and electric fields

**Supplementary Information:**

The online version contains supplementary material available at https://doi.org/10.1140/epje/s10189-026-00632-5.

## Introduction

Controlling the wettability of solid surfaces is a central challenge in interface science, with far-reaching implications for a range of applications [1]. In particular, the ability to dynamically tune liquid–solid interactions plays an important role in microfluidic devices [2–6], lab-on-a-chip systems [7], microreactors [8, 9], adaptive coatings [10] and self-cleaning or anti-fouling surfaces [9]. Precise manipulation of contact angles can enable controlled transport, mixing, and confinement of small liquid volumes, which is a central part for functional integration in fluidic platforms [11].

Among the available approaches, electrowetting (EW) is a well-established and versatile tool for actively modulating surface wettability by applying an electric potential [12–15]. However, despite being a powerful approach to control wetting on surfaces, usage of EW is to some extend also impaired by associated challenges. That is, the need for application of a high electric potentials (up to ~100 V) in order to modulate wetting of thicker dielectric layers on surfaces. In addition, application of high potentials also can cause unwanted side reactions such as electrolysis and degradation [16]. More recently, the complementary concept of electrodewetting (EDeW) [17–21] was introduced and provides additional means to reversibly alter wettability in the opposite direction, thereby expanding the range of (electro)chemical possibilities to remotely control surface wetting. In EDeW approaches, both small electric currents and comparatively small potentials are needed to drive the systems effectively and to cause larger contact angles [20, 21]. That is, because EDeW relies on electric field-induced manipulation of surfactant-laden drops which are deposited on thin hydrophilic oxide layers (~ 2 nm) on a conductive substrate i.e., highly doped silicon wafers with a natively grown surface oxide. On the molecular scale, the mechanism of the EDeW was previously investigated using vibrational sum-frequency generation (SFG) spectroscopy and imaging ellipsometry [21]. According to the proposed mechanism, application of a potential leads to electrophoresis of ionic surfactants such as dodecyltrimethylammonium bromide (DTAB) which can migrate along the water–vapor interface toward the 3-phase contact line. Upon reaching the 3-phase contact line, they spread to the adjacent solid–vapor interface and cause an increase in the apparent contact angle. Although both EW and EDeW strategies offer fast response times and straightforward integration into device architectures, they typically require direct electrical contact and external circuitry.

Soft substrates that can respond upon external stimuli, react, or deform in the presence of a wetting fluid and thereby change their properties have attracted attention in recent years. Note that often also different terminologies like driven, reactive as well as active wetting have been introduced to describe the apparent effects as was recently discussed by Thiele [22]. In particular, soft polymer surfaces are prominent examples where both detailed experiments and simulations have been performed in order to elucidate wetting dynamics as well as condensation on such surfaces [23–31]. In parallel, alternative strategies have emerged that enable remote but non-contact control over wettability. Notably, photoswitchable self-assembled monolayers (SAM) i.e., based on azobenzene [32–34], diazocine [35], or arylazopyrazole (AAP) [36–38] derivatives allow reversible changes of surface properties, but also approaches with thin polymer films containing photoswitchable moieties such as spiropyran [39, 40] or AAP [41] derivatives as well as polymer brushes [30, 31, 42] that can be responsive to different stimuli have been reported. Light irradiation with visible and UV light is often used to trigger E/Z photoisomerization or ring opening/closing reactions which can cause both structural changes, changes in dipole moment and the ability to form hydrogen bonds of the switchable layer with interfacial water molecules [38]. Thus, photoswitchable SAMs can exploit light-induced conformational changes at the molecular level to alter surface energies and, consequently, the apparent contact angle of liquid droplets. While such photoresponsive surfaces have demonstrated reversible changes in static contact angles even with droplets remaining in place, their practical applicability is often limited by pronounced contact angle hysteresis [32, 37, 43]. In many cases, the hysteresis exceeds the magnitude of the light-induced wettability change and can effectively suppress reversible switching, which restricts the system to a unidirectional response [36, 37]. To overcome this limitation of photoswitchable SAMs remains still as a key challenge in the development of truly reversible light-switchable systems, enabling controlled and reversible changes in surface wetting.

**Fig. 1:**
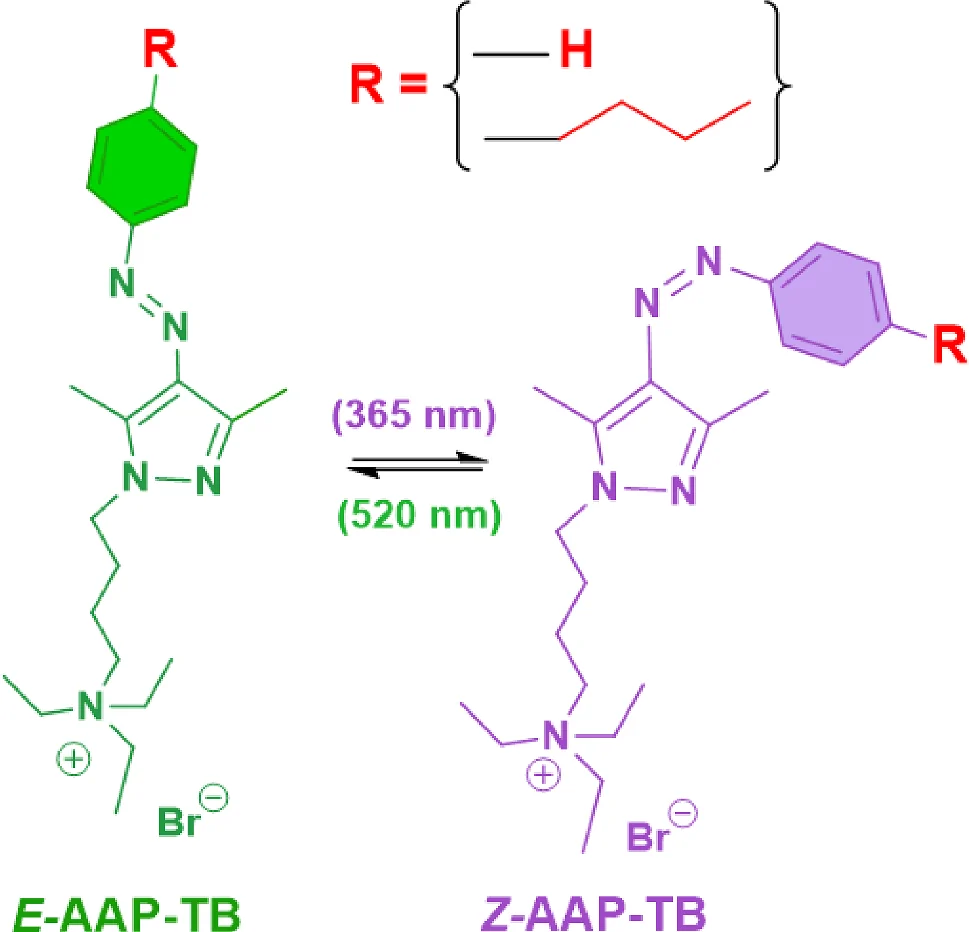
Chemical structures of amphiphilic arylazopyrazole-triethylammonium bromides C_0_AAP-TB and C_4_AAP-TB in their *E* (green color) and *Z* (purple color) configurations which can be prepared by irradiation with 520 nm green and 365 nm UV light, respectively

In this work, we present a conceptually new approach to electrodewetting by integrating potential control with molecular photoswitching. Building on established strategies that employ classical ionic surfactants such as dodecyltrimethylammonium bromide (DTAB) or sodium dodecyl sulfate (SDS) to modulate interfacial tensions under applied potentials [20, 21], we replace these conventional surfactants with photoswitchable AAP amphiphiles. The AAPs used in our work (Fig. 1) exhibit pronounced and reversible changes in surface tension upon *E*/*Z* photoisomerization, combined with exceptionally high photostationary states of >90% in both switching directions [44–46]. Moreover, the *Z* isomer is characterized by long thermal lifetimes ranging from hours to weeks if the pH of the aqueous solution is not too low [47, 48]. By combining these properties, we introduce a dual-responsive system combining light and electric potentials as triggers to control the surface wettability. Specifically, *E*/*Z* photoisomerization of the surfactant provides a means to change interface tensions, while applying an external electric potential induces electrodewetting. This coupled approach allows modulation of the apparent contact angle beyond what is achievable by either stimulus alone, offering enhanced control over droplet behavior at solid–liquid interfaces.

## Experimental details

### Sample preparation

In this study, 10 mm × 10 mm conductive substrates cut from a p-doped Si(100) wafer (resistivity ≤ 0.001 Ωcm; Okmetic) were used with their native oxide layer for all wetting experiments. Prior to each experiment, the substrates were cleaned in piranha solution [3:1 of H_2_SO_4_ (98%) and H_2_O_2_ (35%)] for at least 30 min at a temperature of 80 °C. This was followed by rinsing the substrates with copious amounts of ultrapure water (total organic carbon ≤ 5 ppb, 18.2 Ωcm) taken from a Millipore Reference A+ purification system. Subsequently, the substrates were dried in a stream of nitrogen gas (99.999%, Westfalen) and an additional air plasma treatment was done for about 20 min in order to further activate the oxide layer and to make it even more hydrophilic. The synthesis procedure for the photosurfactant arylazopyrazole-triethylammonium bromide (C_0_AAP-TB) was as described previously by Schnurbus et al. [49], while the butyl-arylazopyrazole-triethylammonium bromide (C_4_AAP-TB) surfactant was synthesized as reported in the Supporting Information. In order to avoid autophobing [50, 51] of the silicon oxide surface through electrostatically favored adsorption of the cationic surfactants due to the negatively charge silicon oxide surface (in case of pH >2.5), the pH of all solutions used was lowered. For that, all solutions were adjusted to pH 2.2 by adding aliquots of hydrochloric acid (30%, Merck Suprapur). Note, that at pH ~2.2, the Si/SiO_2_ surface remains nearly charge neutral, which can effectively mitigate the unwanted autophobing of the silicon oxide surface [20]. Further, both the decrease in the pH as well as the addition of ionic surfactants causes an increase in the ionic strength of the solutions between 6.8 and 13.3 mM for C_0_AAP-TB, and between 6.4 and 6.8 mM for C_4_AAP-TB, which is necessarily dependent on surfactant concentration and is detailed in the Supporting Information. All liquid solutions were used as prepared and were not degassed prior to use. All measurements were conducted under ambient laboratory conditions at 295 K room temperature.

### Contact angle goniometry

Apparent contact angles were measured using a Krüss DSA 100 goniometer which was operated in a sessile drop configuration. A drop of the AAP solutions with a volume 3 µL was placed onto the freshly cleaned Si/SiO_2_ surface using a micro-syringe. A flame-annealed platinum wire (ChemPur, 99.999%) with a diameter of 0.25 mm was employed as an electrode. The electrode was inserted into the droplet while avoiding direct contact with the substrate surface. The substrate was positioned on a silver-painted (SPC, Electrolube) sample holder which had a honeycomb structure filled with ultrapure water in order to maintain a humid vapor environment during EDeW. Another platinum wire (ChemPur, 99.999%) with a diameter of 0.25 mm was connected to a sample holder (with silver paint) to ensure electric contact with the substrate. Both Pt wires were connected to a VersaSTAT3 potentiostat (Princeton) that was used as a source to apply an electric potential while also measuring a current flow between these two electrodes. The contact angles of the sessile drops were recorded and are reported as apparent contact angles in the spirit of Young that are outside the range of any microscopic molecular interaction forces [52]. Below we will often use the term ‘the contact angle’ which always means the apparent contact angle. *E*/*Z* photoisomerization of the dissolved C_0_AAP-TB and C_4_AAP-TB surfactants (Fig. 1) was done in situ during EDeW experiments using two LEDs with emission wavelengths of 520 nm (green) and 365 nm (UV). The LEDs were mounted to the closed sample cell which is described above. For the experiments, the light intensities were adjusted at the sample position to ~2.5 and ~7.5 mW/cm^−2^ for green and UV light, respectively. The reported contact angles are based on at least six independent experiments which were carried out according to the above-described preparation protocol.

### Surface tensiometry of cationic arylazopyrazole derivatives

Surface tension γ isotherms for adsorption of C_0_AAP-TB and C_4_AAP-TB surfactants to the water–vapor interface were determined through drop-shape analysis using a pendant drop tensiometer (PAT-1 M, Sinterface). The tensiometer was equipped with a measurement cell that had two long-pass colored glass filters (FGL590, Thorlabs) as windows to prevent wavelengths <590 nm i.e., from ambient room light and the tensiometers light source to interact with the photoswitchable surfactants. The LEDs were mounted to the sides of the cell, perpendicular to the optical axis of the tensiometer in order to facilitate irradiation of the pendant drop and, thus, *E*/*Z* photoisomerization while the dynamic surface tension was continuously monitored. For tensiometry, dilutions of AAP-TB surfactants in ultrapure water were prepared from 100 and 10 mM stock solutions for C_0_AAP-TB and C_4_AAP-TB, respectively. Using pendant drop tensiometry, dynamic surface tensions were measured for at least one hour while the drop was irradiated with green light (520 nm, 2.5 mW/cm^−2^). Once equilibrium was reached, the light irradiation was switched in situ from green light to UV (365 nm, 5 mW/cm^−2^) to induce *E* →Z photoisomerization, while measurements of the dynamic surface tension were continued without interruption. The equilibrium surface tensions reported below represent the average of at least 20 consecutive data points collected after the surface tension had reached a stable plateau. The overall experimental variation across the entire isotherm was within ±1 mN/m.

## Result and discussion

**Fig. 2:**
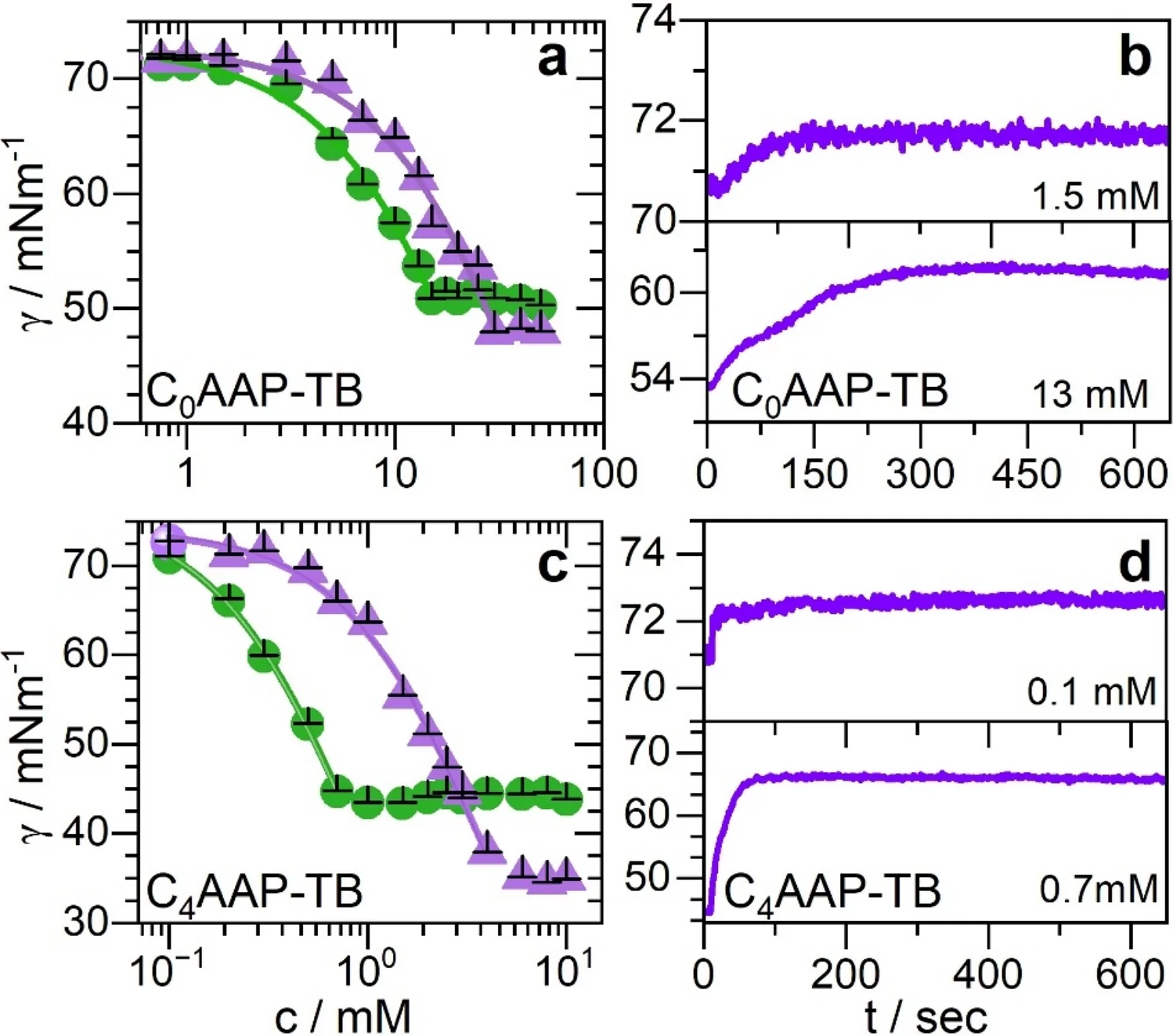
Equilibrium surface tensions *γ* of **a** C_0_AAP-TB and **c** C_4_AAP-TB photoswitchable surfactants at the water–vapor interface as a function of their bulk concentrations. Note that the surface tensions were recorded under continuous irradiation with either green light at wavelength of 520 nm (2.5 mW/cm^−2^) or UV light at 365 nm (5 mW/cm^−2^) in order to prepare the predominant *E* or *Z* isomers of both surfactants (Fig. 1, Table S1). Results for the *E* isomers are shown as green circles while results for the *Z* isomers are indicated as purple circles. Solid lines guide the eye. Figures **b** and **d** show the dynamic surface tension $\gamma \left(t\right)$ after the surface was equilibrated under green light and the UV light was switched on at *t*=0 s, where **a** presents $\gamma \left(t\right)$ for C_0_AAP-TB at 1.5 and 13 mM molar concentration and **b** for C_4_AAP-TB at 0.1 and 0.7 mM concentration

In this work, we address the EDeW behavior in the presence of photoswitchable amphiphiles (Fig. 1) which can undergo efficient *E*/*Z* photoisomerization with and can, thus, change their interfacial properties. For this purpose, we will initially investigate how the C_0_AAP-TB and C_4_AAP-TB surfactants, with different terminal alkyl chains, behave at the water–vapor interface as a function of their bulk concentration. Previous studies have demonstrated that AAP derivatives can photoisomerize into their predominant *E* configuration under irradiation with green light, while UV irradiation drives them into the Z configuration. At neutral pH, their photostationary states are >95% for *E*/*Z* switching in both directions enabling near quantitative switching (Supporting Information, Table S2). The thermal lifetime of the *Z* isomer for spontaneous *Z* → *E* back-isomerization is for AAP derivatives at neutral pH values with many hours (Figure S7b) comparatively long and superior to most azo derivatives. However, at low pH values which we have applied in this work, the thermal lifetime of the *Z* isomer is drastically reduced as was recently reported by Ludwanowski et al. [47] using a similar AAP derivative. Indeed, for our surfactants the lifetimes of the *Z* isomers are only ~ 84 ± 0.1 s for C_0_AAP-TB and ~10 ± 0.1 min for C_4_AAP-TB at the low pH value of 2.2 used in this study (more details are given in the SI). Thus, constant UV irradiation is needed to establish photostationary states of >90% *Z* (SI). Since all experiments were performed in situ under constant irradiation of either green (520 nm) or UV light (365 nm), only differences in photostationary states between *E* and *Z* isomers are important for our interpretations below. The change in interfacial properties was studied by pendant drop tensiometry and the results for the equilibrium surface tensions for C_0_AAP-TB and C_4_AAP-TB surfactants in their *E* as well as *Z* configurations at the water–vapor interface are presented in Fig. 2a and c.

From the surface tension isotherms shown in Fig. 2a and c, the CMC of both the *E* and *Z* isomers can be determined. CMC values of both C_0_AAP-TB and C_4_AAP-TB surfactants, can be identified by the apparent sharp kinks in the surface tension isotherms which are followed by an extended plateau where the surface tension becomes independent of the bulk concentration. As shown in Fig. 2a and c, the surface tension isotherms differ for both surfactants substantially and are also different when their *E* and *Z* isomers are compared. From Fig. 2a, we determine the CMC of the C_0_AAP-TB surfactant to 15 and 30 mM for continuous irradiation with green (predominant *E* isomers) and UV (predominant *Z* isomers) light, respectively. This is in case of C_0_AAP-TB in excellent agreement with previous results [49], while C_4_AAP-TB surfactants have been synthesized for this study and, thus, comparison with previous work is not possible.

In case of C_4_AAP-TB, the additional terminal-butyl chain at the AAP center renders the surfactants more hydrophobic and shifts the CMC of both *E* and *Z* isomers generally to lower concentrations (Fig. 2). As a result, the CMCs for *E* and *Z* isomers of C_4_AAP-TB are located at 0.75 and 5 mM, respectively (Fig. 2c). Furthermore, a close comparison of the isotherms in Fig. 2a and c for predominant *E* and *Z* isomers (corresponding to irradiation with green and UV light) shows differences in the surface tension between predominant *E* and *Z* isomers, when the concentration is ≤ CMC. Particularly, for C_4_AAP-TB dramatic changes in the surface tension between *E* and *Z* isomers are observed at CMC of *E* isomer (0.75 mM). In this case, the surface tension of the water–vapor interface changes drastically by 20 mN/m upon *E*/*Z* photoisomerization (Fig. 2a). This is consistent with a similar but anionic photoswitchable AAP derivative reported elsewhere [48, 53, 54]. For C_0_AAP-TB surfactants, *E*/*Z* photoisomerization at a fixed concentration, i.e., 15 mM, also causes changes in surface tension (Fig. 2a). These changes are, however, with a change of ~8 mN/m much less pronounced. Clearly, we can make use of the C_0_AAP-TB and C_4_AAP-TB surfactants to remotely trigger changes at the water–vapor interface and likely also at other interfaces relevant for wetting of a surfactant-laden drops at a solid substrate.

As we are also interested in the dynamic properties of the water–vapor interface, we additionally plot the dynamic surface tension, γ(t), in Fig. 2b and c. These panels compare the evolution of the surface tension following equilibration under UV irradiation and subsequent switching to UV light to induce the *E* → *Z* isomerization. For Fig. 2b, C_0_AAP-TB concentrations of 0.7 and 13 mM were selected, whereas for the more surface-active C_4_AAP-TB, concentrations of 0.1 and 1.5 mM are shown. As evident from Fig. 2b and c, the new interfacial equilibrium state, dominated by the *Z* isomer, is reached within less than 150 s for all concentrations. As we will discuss below (Fig. 4), this time scale closely matches that of the contact angle change observed for droplets containing our photoswitchable surfactants.

**Fig. 3:**
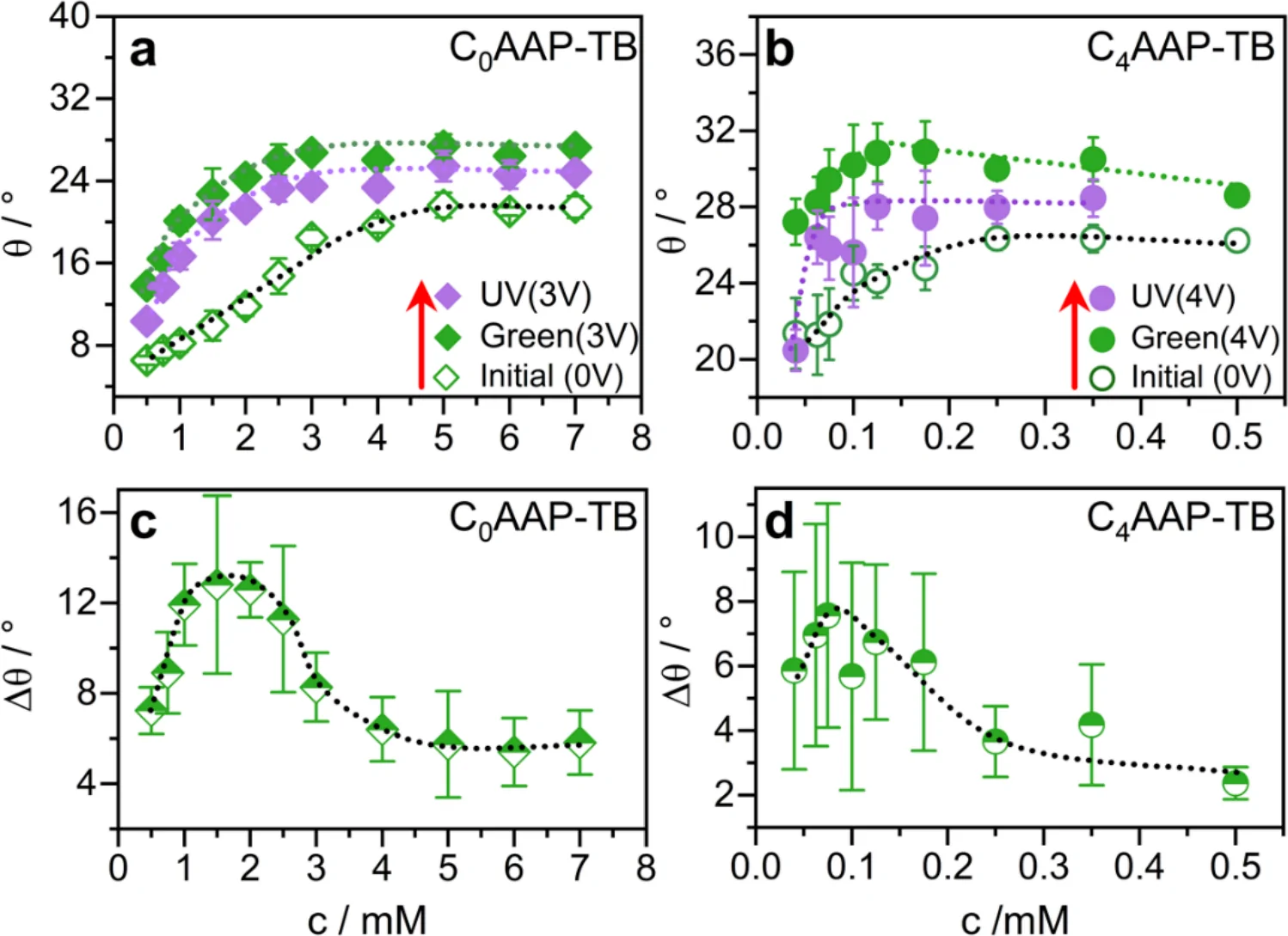
Contact angles of surfactant-laden drops (3 µl) for different concentrations of **a** C_0_AAP-TB and **b** C_4_AAP-TB surfactants. For this, the solution pH was fixed to 2.2 and drops were continuously irradiated with 520 nm green light to prepare the *E* isomer or with 365 nm UV light for the *Z* isomer. As indicated in the figure contact angles at different potentials 0 V (open diamonds) as well as for +3 V or + 4 V are shown for **a** C_0_AAP-TB and **b** C_4_AAP-TB surfactants. In **c** the contact angle difference from **a** between the contact angles at +3 V and 0 V are shown while **d** presents the difference between contact angles from **b** between +4 V and 0 V. Red arrows in **a** and **b** indicate the which order the changes were done starting from the initial wetted state at 0 V with predominant *E* isomers, to the dewetted state at an applied potential (+ 3 V or +4 V) and the, subsequently, the changes to the *Z* isomer through UV irradiation. Dashed lines in guide the eye

Next, we will apply the above-described effects at the water–vapor interface to tune surface tensions and, consequently, the apparent contact angle using *E*/*Z* photoisomerization during EDeW of a surface. For this purpose, we recorded the contact angle of aqueous drops at pH 2.2 with varying surfactant concentrations. We recall, that the low pH was chosen to prevent autophobing of the terminating silicon oxide surface in contact with an aqueous electrolyte that contains cationic surfactants. For the measurements, drops were first deposited onto the conductive silicon substrates with native oxide layers, and the initial contact angle was recorded without applying a potential (0 V). This was performed under continuous irradiation with green light to ensure that the surfactants were predominantly in their more surface-active E form (see Fig. 2). Subsequently, a potential of +3 V was applied, which induced dewetting of the surface and led to an increase in the contact angle (Fig. 3). This change in contact angle Δ*θ*, defined as the difference in contact angle between the dewetted (EDeW) state at +3V and the initial wetted state at 0 V, depended on the surfactant concentration. This observation is fully consistent with previous studies which investigated classical surfactants such as DTAB or SDS [20, 21]. From Fig. 3a and b, which shows the initial contact angles (0 V) and the contact angles in the dewetted state for the E isomers of C_0_AAP-TB (+3 V) and C_4_AAP-TB (+4 V) as a function of concentration, a similar trend is observed, although, the concentrations differ by approximately one order of magnitude which is again related to the different surface activities of the two surfactants. At low concentrations, the initial contact angles increase with concentration and reach a plateau at approximately one-third of the respective CMC values. Beyond this point, the contact angle becomes concentration-independent—an effect that is similar for both C_0_AAP-TB and C_4_AAP-TB (Fig. 3). Note, that we will explain the reason why different potentials were chosen for C_0_AAP-TB and C_4_AAP-TB surfactants below, but will now focus on the concentration dependence.

After applying, a sufficiently positive potential, while the surfactants were kept in their E form, a qualitatively similar trend with concentration is observed, but all contact angles are systematically larger indicating dewetting of the aqueous drop from the substrate surface. In Fig. 3c and d, we quantify the EDeW by showing the contact angle change Δ*θ*, as defined above, versus concentration. Clearly, Δ*θ* shows large variations with surfactant concentration with a pronounced maximum at about 1/10 of the respective CMCs of C_0_AAP-TB and C_8_AAP-TB. As a comparison with previous works on EDeW shows, the trend in Δ*θ* with varying concentration is largely independent of the chemical nature of the ionic surfactant.

However, at this point we recall the proposed mechanism of EDeW is requiring not only the application of a potential but also a small but nonzero electric current (see Supporting Information) between the wire immersed in the drop and the substrate [20, 21]. This behavior was explained by electrophoresis of mobile ionic surfactants, which migrate along the water–vapor interface toward the 3-phase contact line. At the 3-phase contact line, they are deposited and drive dewetting of the substrate surface. Particularly, the electrophoresis of ionic surfactant depends on the bulk concentration and the surface excess of surfactants. At low concentrations, the surface coverage is too small to generate significant interfacial tension gradients, whereas at higher concentrations (approaching the CMC) the interface becomes densely packed, limiting lateral mobility of the surfactants. Consequently, at intermediate concentrations, which are well below the CMC, there is an optimal balance between sufficient surface excess and interfacial mobility, resulting in the observed maximum in Δ*θ*. In addition to the latter, transport of ions and surfactant in the bulk solution also contributes to the effect and largely depends on the conductivity of the aqueous electrolyte. At this point, we want to elaborate more on why the maximum in Δ*θ* is found at concentrations close to 1/10 of the CMC independent on the chemical nature of the surfactant. For that, we are discussing the role surface coverage and the conductivity of the surfactant solution. For that, we describe the adsorption surfactants to the water–vapor interface with the Langmuir isotherm [55]

1 $$\alpha =\Gamma /{\Gamma}_{\mathrm{m}\mathrm{a}\mathrm{x}} =\frac{\mathrm{K}\mathrm{C}}{1+\mathrm{K}\mathrm{C}} \Rightarrow \mathrm{K}\mathrm{C}=\frac{\alpha }{1-\alpha }$$

where C is the surfactant bulk concentration, K the equilibrium constant for adsorption, and Γ as well as ${\Gamma}_{\mathrm{m}\mathrm{a}\mathrm{x}}$ are the surface excess a given concentration and the maximum surface excess, respectively. At the CMC usually only a fraction ${\alpha}_{\mathrm{m}\mathrm{a}\mathrm{x}}$ (roughly between 0.90 and 0.95) of the theoretical maximum surface coverage α=1 can be reached [52]. This largely depends on the Gibbs free energy for interface adsorption and micelle formation [56]. In order to gain an empirical model, we implement our observation that the response function $R\left(c\right)$ for contact angle changes under EDeW depends on: (i) the conductivity of the wetting drop $\sigma \approx {\Lambda}_{m}^{0}C-\mathcal{K}{C}^\frac{3}{2}$ with ${\Lambda}_{m}^{0}$ the limiting molar conductivity and $\mathcal{K}$ the Kohlrausch constant. (ii) The ability of the surface to change its coverage as a function of concentration $\left(\frac{\partial \alpha }{\partial C}\right)$. For the conductivity we assume a strong electrolyte in a, however, very dilute regime justifiable by the low concentrations (< 1 mM) where we observe the maximum in Δ*θ*. Consequently, we approximate Kohlrausch law as shown above and only consider the linear term. Thus, we write for the response function

2 $$R\left(c\right)\propto {\Lambda}_{m}^{0}C \left(\frac{d\alpha }{dC }\right)=\frac{\mathrm{K}\mathrm{C}}{{\left(1+\mathrm{K}\mathrm{C}\right)}^{2}}$$

Using the derivative of the latter

3 $$\frac{dR}{dC}\propto \frac{K-C{K}^{2}}{{\left(1+\mathrm{K}\mathrm{C}\right)}^{3}}$$

We find the maximum of the responsiveness where we can also use Eq. (1) to find a dependence between the surfactant concentration ${C}_{\mathrm{m}\mathrm{a}\mathrm{x}}$ with the maximum $R\left(C\right)$ in EDeW and the CMC:

4 $${C}_{\mathrm{m}\mathrm{a}\mathrm{x}}=\frac{1}{K}={C}_{CMC}\frac{{1-\alpha }_{\mathrm{m}\mathrm{a}\mathrm{x}}}{{\alpha}_{\mathrm{m}\mathrm{a}\mathrm{x}}}$$

Clearly, this is independent on the exact chemical nature of the surfactant. In case, the maximum surface overage ${\alpha}_{\mathrm{m}\mathrm{a}\mathrm{x}}$ ranges between 0.90 and 0.95, ${C}_{\mathrm{m}\mathrm{a}\mathrm{x}}$ varies between ~0.11 and 0.05 CMC, which is consistent with the above-described experimental results and reported data in previous works [20, 21].

From a close comparison of Fig. 3c and d, it can be observed that the overall shape of Δ*θ* as a function of concentration is very similar for both surfactants, but the absolute values of the contact angle change Δ*θ* differ by about a factor of two and are larger for C_0_AAP-TB. We propose that these differences are likely correlated with the electrolyte concentration in the studied systems. Since the required concentrations of C_0_AAP-TB surfactants are ~10 times higher than those of C_4_AAP-TB, using C_0_AAP-TB leads to a higher conductivity and also to a higher ionic strength. This improves charge transport in the drop and reduces resistive losses, so that lower potentials can be applied and are sufficient to drive the EDeW response. In contrast, for C_4_AAP-TB the lower electrolyte concentration results in reduced conductivity, requiring the application of a larger potential (+ 4 V) to achieve comparable effects in EDeW (see also Figure S9 in the SI). We can therefore conclude that for EDeW, C_0_AAP-TB is the more effective surfactant under the present conditions, as the higher ionic strength enhances charge transport and supports larger interfacial tension gradients and, consequently, larger Δ*θ*.

Having discussed EDeW with C_0_AAP-TB and C_4_AAP-TB surfactants, we now turn to contact angle changes as well as drop splitting driven by *E*/*Z* photoisomerization. Previously, Zhang et al. [57] investigated the wetting behavior of silica surfaces using a cationic photoswitchable azo surfactant. Upon switching from visible to UV light, they observed a decrease in contact by ~7°. These experiments were conducted at pH 7, where electrostatically favored adsorption of the cationic azo switches onto the negatively charged silica surface occurs. Zhang et al. attributed the reduced contact angle to significant desorption upon UV irradiation at the water–vapor and the solid–water interface. In contrast, our experiments were conducted at pH 2.2 to suppress deprotonation of the silanol groups at the silicon oxide surface. This results in a nearly charge-neutral surface and minimizes the otherwise electrostatically favored adsorption of the cationic AAP surfactants. Clearly, the choice of pH 2.2 can reduce the influence of adsorption to the solid–liquid interface and, in particular, can mitigate autophobing effects. At this point, it also important to note that AAP surfactants can undergo protonation at the exposed azo group of the Z isomers, which was previously shown by Walther and co-workers [47]. In their work using different AAP derivatives a pK_a_ of ~1.6 was determined, which is similar to what we find for our surfactants as shown in detail in the SI. Consequently, at pH 2.2 a small fraction (≤ 20%) of cationic AAP surfactants is expected to be protonated at the azo group resulting in molecules that carry two positive charges rather than only a single positive charge from the trimethylammonium headgroup (SI). However, this additional protonation is not expected to significantly affect the interaction with either the water–vapor or the neutral silica surface.

Building on the above discussed results for the initial and dewetted states with the AAP surfactants in the *E* configuration, we further investigated the effect of *E* → *Z* photoisomerization through continuous UV irradiation while maintaining a constant potential (+ 3 V/+4 V). The resulting contact angles are shown in Fig. 3a and b together with the contact angles of initial and dewetted states with predominant *E* isomers in the drop. For the concentrations studied, switching to the *Z* isomer led to a systematic decrease in contact angle. For C_0_AAP-TB, the reduction is approximately 3°, whereas for C_4_AAP-TB it is with ~6° more pronounced. These differences can be partially attributed to the corresponding changes in surface tension at the water–vapor interface as indicated in Fig. 2 and discussed above. In terms of surface tension changes, C_4_AAP-TB exhibits larger variations than C_0_AAP-TB. Overall, E/Z photoswitching of interfacial AAP moieties enables fine-tuning of the contact angle during EDeW. However, the magnitude of this effect is limited by the low surfactant concentrations required for EDeW, typically around 1/10 of the CMC. As shown in Fig. 2, differences in surface tension between E and Z isomers are significantly smaller at these low concentrations, while near the CMC the photoswitchable surfactants generally exhibit their largest interfacial effects upon *E*/*Z* photoisomerization.

**Fig. 4:**
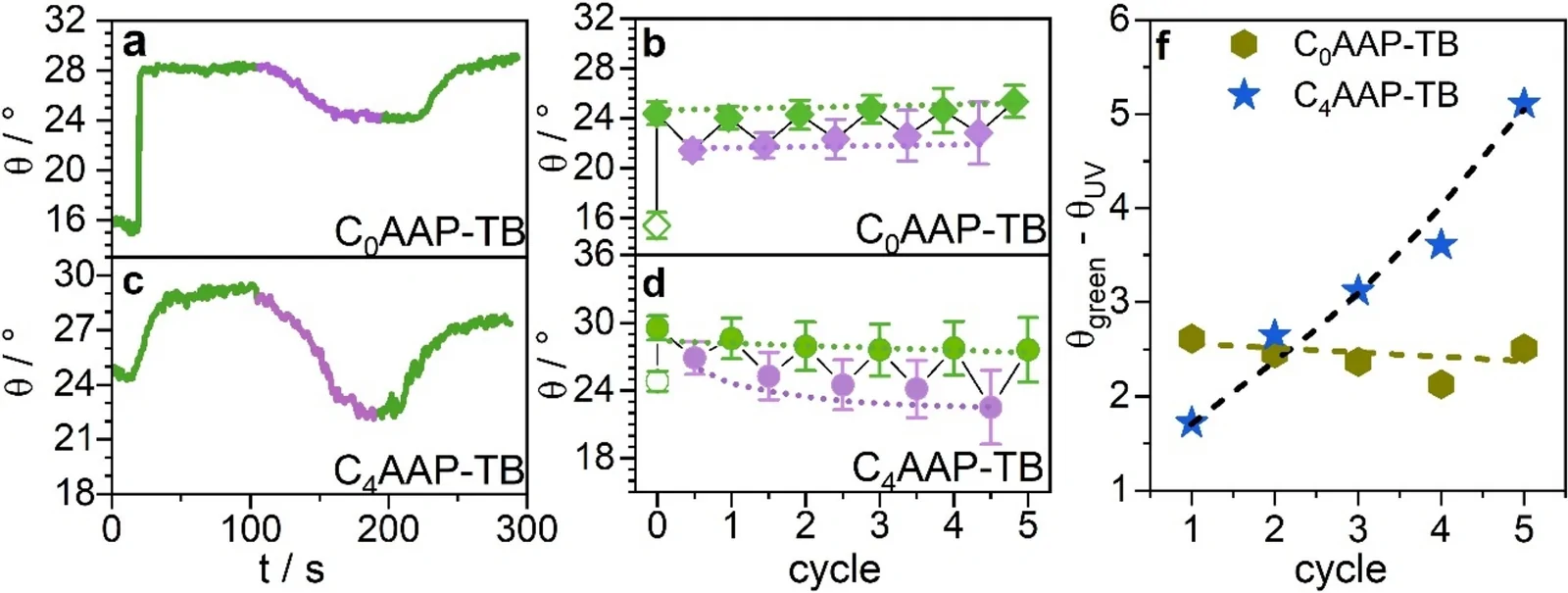
Contact angle of aqueous drops containing **a** and **b** 2 mM C_0_AAP-TB as well as **c** and **d** 0.1 mM C_4_AAP-TB at pH 2.2. **a** and **c** show the dynamic contact angles during electrowetting and subsequent *E*/*Z* photoisomerization with green color indicating predominant *E* isomers while purple color indicates the change to predominant *Z* isomers. **b** and **d** demonstrates the changes in contact angles after different *E*/*Z* photoisomerization cycles. Open symbols in **b** and **d** indicate the initial contact angle at 0 V, while filled symbols show the observed contact angles at a potential of +3 V (**b**) and +4 V (**d**) upon irradiation at 520 nm green (*E* state) and 365 nm UV light (*Z* state). **f** Displays the difference in contact angle ($\Delta \theta ={\theta}_{\mathrm{g}\mathrm{r}\mathrm{e}\mathrm{e}\mathrm{n}}-{\theta}_{\mathrm{U}\mathrm{V}}$) between over five switching cycles for C_0_AAP-TB (hexagons) and C_4_AAP-TB (stars). Dashed lines in **b**, **d** and **f** guide the eye

In Fig. 4, we present results for the contact angle as a function of repeated switching cycles between *E* and *Z* states for both C_0_AAP-TB and C_4_AAP-TB surfactants, which clearly indicate that the observed change in contact angle is highly reversible. In fact, the dynamic contact angles in Fig. 4a (C_0_AAP-TB) and 4c (C_4_AAP-TB) are also showing the kinetic change when switching from the predominant *E* to the *Z* isomer. Switching in either direction results in similar rates of the contact angle change. This is substantially different for most photoswitchable self-assembled monolayers containing either AAP or Azo derivatives on a solid substrate. Here often a large contact angle hysteresis is observed and impairs reversible switching of the layer while the drop is kept in contact with the surface [27, 28]. Only in exceptional cases such monolayers have sufficiently low contact angle hysteresis enabling reversible switching and even light propelled motion of drops [32, 43].

Figure 4a and c also shows that the time scale of the contact angle changes is very similar to the time scale of the changes in dynamic surface tension (see Fig. 2c and d). We can therefore hypothesize that the changes in contact angle are driven by change in surface tension at the water–vapor interface. In addition, the contact angle differences ($\Delta \theta$) between surfactants in their dominant *E* (green) and *Z* (UV) conformation as shown in Fig. 4f, do also reveal differences. For C_0_AAP-TB, the differences between consecutive *E*/*Z* switching cycles are neglectable, while for C_4_AAP-TB a progressive increase of the contact angle difference is seen and is likely related to the more substantial spreading of the surfactant beyond the 3-phase contact line as well as to the (expected) more drastic changes in surface tension of C_4_AAP-TB.

**Fig. 5:**
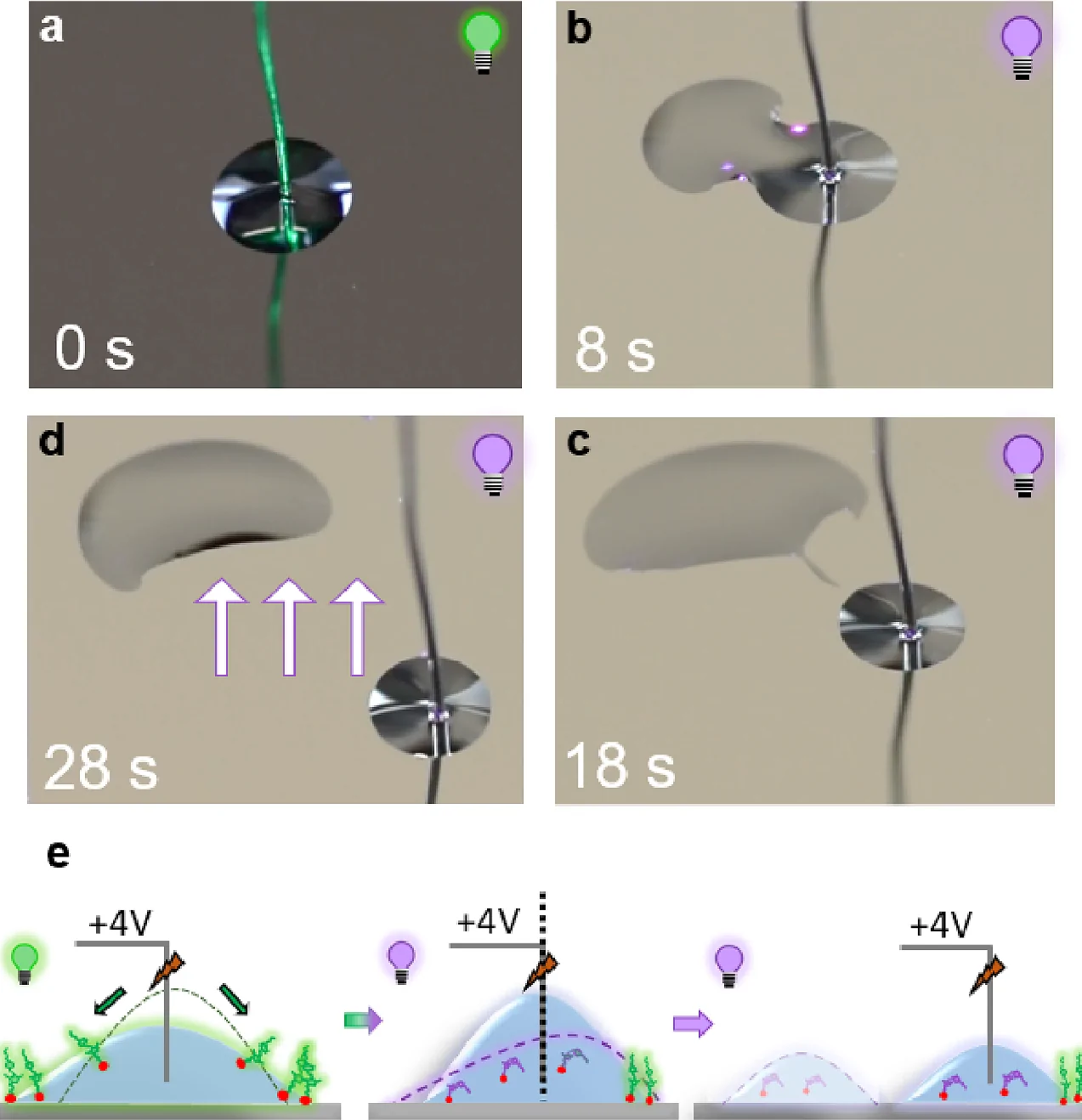
**a** First, a 3 µl C_4_AAP-TB drop was irradiated with 520 nm green light at 2.5 mW/cm^−2^ intensity while a potential of +4 V was applied to drive EDeW. At times >0 s, the irradiation was changed to very intense UV light (365 nm with 11.5 mW/cm^−2^) and photograph shown in **b**, **c,** and **d** are taken at later times as indicated in the figure. A video of this experiment is supplied as Supplementary Information. **e** Shows a schematic of the splitting and the moving process for the C_4_AAP-TB containing droplet upon irradiation with the green and the UV light while an external potential is applied. White arrows in **d** indicate the moving direction of the droplet after splitting toward the source of the UV light

After having understood, the overall behavior when applying a positive potential and different light conditions, we will now investigate splitting and directed motion of drops containing the C_4_AAP-TB surfactants. For our investigations, we used a drop with a volume of 3 µL deposited on our substrates and irradiated with green light (2.5 mW cm⁻^2^) while a potential of +4 V was applied (Fig. 5a, 0 s). During this stage, AAP molecules accumulate at the interface and are transported toward the 3-phase contact line during electrodewetting of the surface. Switching from green to comparatively intense UV irradiation (11.5 mW cm⁻^2^) induces rapid interfacial changes. Within ~8 s, a boundary layer forms (Fig. 5b), followed by asymmetric deformation and finally droplet splitting and motion at ~18 s (Fig. 5c). Continued irradiation drives the separated droplet toward the UV light source (~ 28 s, Fig. 5d). This behavior is likely governed by Marangoni flows [48] at the water–vapor interface as well as by diffusio-osmotic flows [58, 59]. Due to inner filter effects, UV light is absorbed non-uniformly within the droplet, leading to spatially heterogeneous *E*→*Z* photoisomerization. This creates a gradient in surfactant *E*/*Z* composition and causes also a gradient in surface tension at the water–vapor interface. The associated flows drive internal convection, droplet deformation, splitting, and subsequent motion toward the irradiated region. Necessarily, the effect is strongly concentration-dependent as at low concentrations (< 0.06 mM), insufficient interfacial coverage prevents the formation of a significant surface tension gradient. At high concentrations (> 0.1 mM), excess surfactant in the bulk dampens interfacial bulk gradients in isomer concentration and suppress both splitting and wettability changes. Thus, the phenomenon is limited to an optimal concentration range within 0.06 to 0.1 mM of C_4_AAP-TB. The higher UV intensity used here (11.5 mW cm⁻^2^, compared with ~7.5 mW cm⁻^2^ for experiments related to Fig. 3) enhances light attenuation within the droplet and amplifies the resulting surface tension gradients. While Marangoni flow and diffusio-osmosis are likely the primary drivers, an additional electrostatic contribution is observed. When the droplet is positioned off-center and UV illumination is switched off during motion, the droplet continues to move. This is attributed to electrostatic attraction between the negatively charged Si/SiO_2_ surface outside the drop where a thin water exists ([21]) and the surfactant-laden droplet. A video of the splitting and motion processes is provided as Supporting Information to this work.

## Conclusion

In this study, we have demonstrated a novel approach to dynamically control wettability by integrating electrodewetting with photoswitchable surfactants. By employing arylazopyrazole-based cationic surfactants, we combined electric-field-driven interfacial transport with light-induced modulation of surface activity. This dual-stimulus concept enables precise and reversible tuning of contact angles beyond the capabilities of either electrodewetting or photoswitching alone. Systematic investigations revealed that both the response in electrodewetting and the magnitude of light-induced effects were strongly depending on surfactant concentration, with an optimal performance observed well below the critical micelle concentration. At these intermediate concentrations, a balance between sufficient interfacial coverage and surfactant mobility facilitates efficient electrophoretic transport and maximizes contact angle changes. While electrodewetting provides the dominant contribution to wettability modulation, *E*/*Z* photoisomerization can be applied to additionally finetune the contact angle, allowing reversible switching with minimal hysteresis. This is compared with many other conventional photoresponsive surfaces an advantage, but it is limited through the low surfactant concentrations where only minor changes upon *E*/*Z* switching at the water–vapor interface are observed. Clearly, overcoming this limitation may even improve the photoresponse for this setup. Moreover, we demonstrated that the coupling of electric fields with spatially non-uniform photoisomerization can generate surface tension and concentration gradients of different isomers that drive surfactant flows and to complex droplet behaviors such as deformation, splitting, and directed motion.

### Supporting information

Further information on synthesis protocol of C_4_AAP-TB surfactants, the measured current during EDeW vs AAPs concentration and the contact angle as a function of different switching cycles, videos showing drop splitting and movement under UV irradiation.

## Supplementary Information

Below is the link to the electronic supplementary material.

Supplementary file1 (PDF 969 KB)

Supplementary file2 (MP4 22380 KB)

## Data Availability

My manuscript has associated data in a data repository. Data will be made available on request.
