# Supplementary material for: Light-gated electro(de)wetting with photoswitchable amphiphiles
Source: Eur Phys J E Soft Matter. 2026 Sep 14;49(9):85. doi: 10.1140/epje/s10189-026-00632-5 (PMC13574899; doi:10.1140/epje/s10189-026-00632-5)
Supplement: Supplementary file 1 [file 10189_2026_632_MOESM1_ESM.pdf]

Supporting Information

Light-Gated Electro(de)wetting with Photoswitchable Amphiphiles

Billura Shakhayeva[1)], *Michael Hardt*[1)] *and Björn Braunschweig*[1)]*

[1)] Institute of Physical Chemistry and Center for Soft Nanoscience, University of Münster,

Corrensstraße 28/30, 48149 Münster, Germany

Corresponding Author *e-mail: braunschweig@uni-muenster.de.

Contents

1. Synthesis of the C_4AAP-TB
2. NMR Spectra
3. Elemental analysis of Arylazopyrazole photoswitches
4. Thermal relaxation dynamics of *Z* – Arylazopyrazole photoswitches
5. pKa value of Arylazopyrazole photoswitches
6. Emission spectra of the LEDs
7. UV/Vis spectra and Photostationary States
8. Calculation of Total Ionic Strength
9. Electric currents and voltage dependance for electro-dewetting

1. Synthesis of the C_4AAP-TB Surfactants

The surfactant synthesis was adapted with minor modifications from our previous works [1–3]. All chemicals were used as received unless otherwise stated.

3-(2-(4-butylphenyl) hydrazono) pentane-2,4-dione

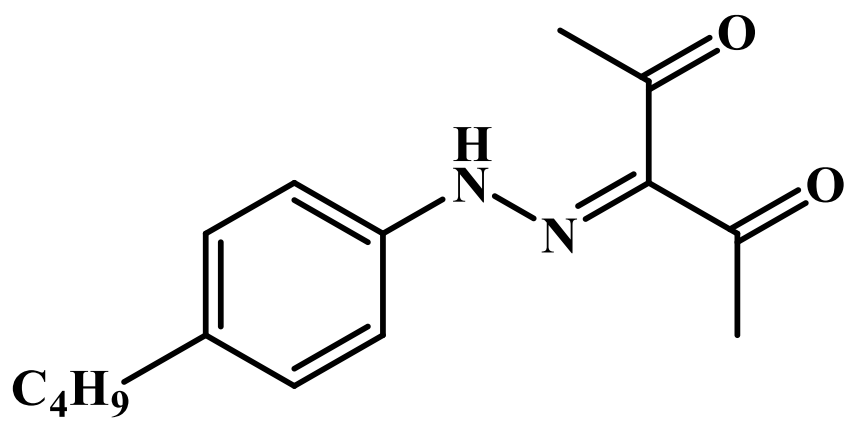

Butyl aniline (9.8 mL, 67.1 mmol, 1.0 eq. – 97%, Acros Organics) was dissolved in a mixture of hydrochloric acid (15.4 mL, 0.23 mL/mmol – 12 M, VWR) and glacial acetic acid (101 mL, 1.5 mL/mmol – 17.4 M, >99.7%, Fisher Scientific). The mixture was cooled down to 0 °C and sodium nitrite (5.6 g, 80.5 mmol, 1.2 eq. dissolved in minimum amount of water – 99.4%, VWR) was added dropwise, without increasing the solution temperature above 5 °C. The reaction mixture was stirred for 1 h at ~0°C. A second mixture containing sodium 2,4-pentandione (9.8 mL, 87.2 mmol, 1.3 eq. – >99%, VWR) and sodium acetate (anhydrous, 16.6 g, 201 mmol, 3 eq. – >99%, chemsolutebio) in ethanol (67.1 mL, 1 mL/mmol – 99.8%, Fisher Scientific) and ultrapure water (38.4 mL, 0.57 mL/mmol) was prepared, and the initial aniline mixture was added slowly to the pentandione mixture. The yellow solid was filtered and subsequently washed with cold ultrapure water, ultrapure water/ethanol (1:1) and cyclohexane. The product was dried under reduced pressure, yielding a yellow solid.

M ($C_{15}H_{20}N_2O_2$): 260.34 g/mol

Yield 91% (15.9 g, 61.2 mmol) as a bright yellow solid

(*E*)-4-((4-butylphenyl)diazenyl)-3,5-dimethyl-1H-pyrazole

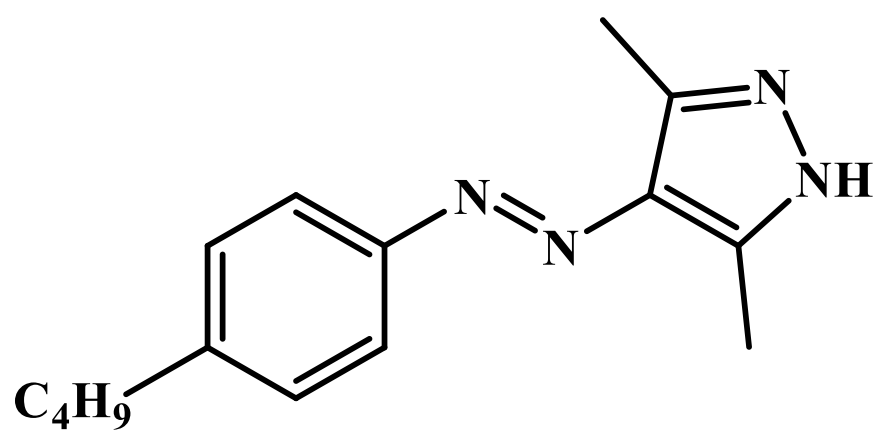

The pentane dione (15.93 g, 61.2 mmol, 1.0 eq) and an aqueous hydrazine solution (5.54 mL, 61.2 mmol, 1.0 eq – 35 wt%, Sigma Aldrich) were dissolved in absolute ethanol (612 mL, 10 mL/mmol – 99.8%, Fisher Scientific) and were stirred for >3 h at 85 °C. Note that the mixture was divided into 8 flasks for the reaction and combined afterwards. The product was obtained as an orange/yellow solid after solvent removal under reduced pressure and used without further purification.

M ($C_{15}H_{20}N_4$): 256.35 g/mol

Yield 95.6% (15.0 g, 58.5 mmol) as a yellow solid

4-bromo-N,N,N-triethylbutan-1-ammonium bromide

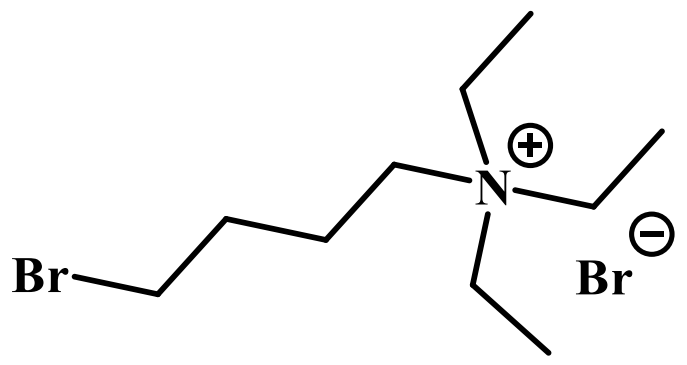

1,4-Dibrombutan (2.8 mL, 23.2 mmol, 1 eq. – >98 %, TCI Deutschland GmbH) and triethylamine (9.2 mL, 23.2 mmol, 1 eq. – PanReac AppliChem) were stirred at 80 °C for >3 h. As the product was too fine for filtration, the reaction mixture was transferred into a pear-shaped flask using methanol (10 mL/mmol – Emsure, Merck KGaA). The solvent was removed by vacuum distillation, without completely drying the product. The remaining product was suspended in ethyl acetate (Merck KGaA) and washed three times by sonicating the suspension, removing the liquid phase and re-suspending in ethyl acetate. The solvent was removed under reduced pressure.

M ($C_{10}H_{23}Br_2N$): 317.11 g/mol

Yield 58% (4.29 g, 13.5 mmol) as a white solid

(*E*)-4-(4-((4-butylphenyl)diazenyl)-3,5-dimethyl-1H-pyrazol-1-yl)-N,N,N-triethylbutan-1-aminium bromide (C_4AAP-TB)

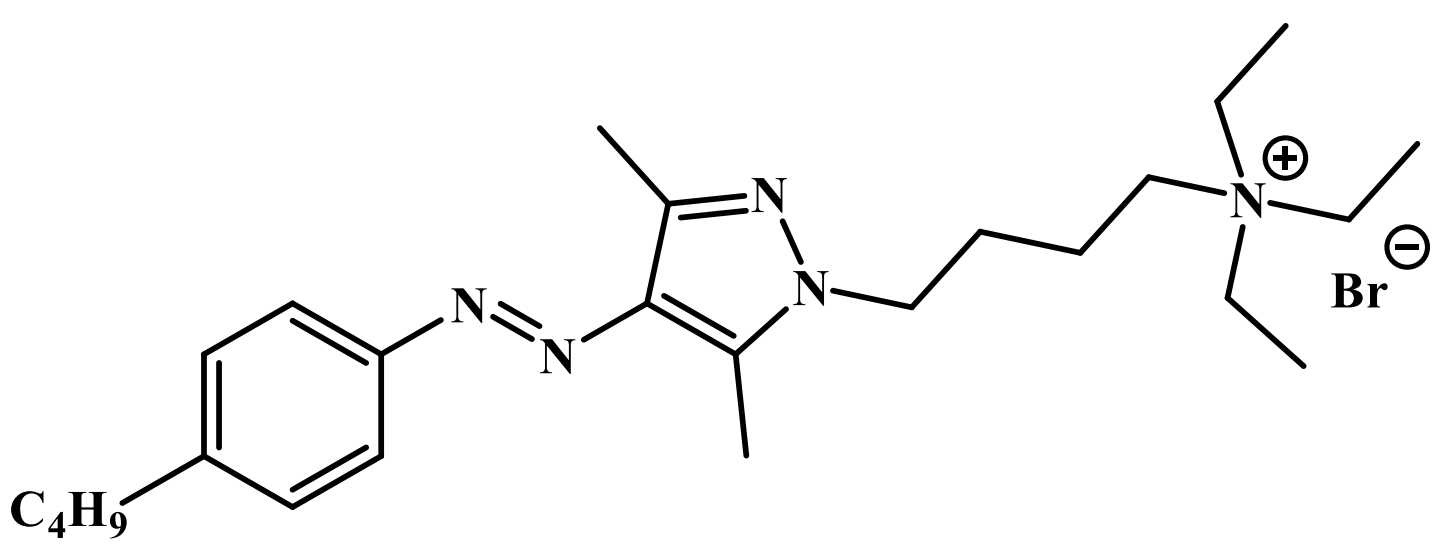

The AAP precursor (2.0 g, 7.8 mmol, 1.5 eq.) was added to a suspension of potassium carbonate (5.4 g, 39.0 mmol, 7.5 eq. – Honeywell) in acetonitrile (101 mL, 13 mL/mmol–Thermo Scientific), and the resulting mixture was stirred for >5 min. The ammonium bromide linker (1.7 g, 5.2 mmol, 1 eq.), dissolved in acetonitrile, was added, and the reaction mixture was refluxed overnight. The remaining potassium carbonate was filtered, and the solvent was removed under reduced pressure. The raw product was suspended and washed multiple times with ethyl acetate until the liquid phase was only slightly colored. The solvent was removed under reduced pressure, and the product was dissolved in acetone. Undissolved particles (mainly from the ammonium bromide linker) were filtered, and the product was dried under reduced pressure. The product was obtained as a yellow solid.

M ($C_{25}H_{42}N_5Br$): 492.55 g/mol

Yield 23% (0.90 g, 1.83 mmol) as a yellow solid

^{1}H-NMR (500 MHz, CDCl3) δ = 7.68 (d, J = 8.4 Hz, 2H), 7.25 (d, J = 8.4 Hz, 2H), 4.16 (t, J = 6.6 Hz, 2H), 3.45 (q, J = 7.3 Hz , 8H), 2.67-2.63 (m, 2H), 2.61 (s, 3H), 2.44 (s, 3H), 2.03 (p, J = 6.9 Hz, 2H), 1.89-1.81 (m, 2H), 1.65-1.58 (m, 2H), 1.40-1.34 (m, 11H), 0.92 (t, J = 7.4 Hz, 3H).

^{13}C{H}-NMR (125.8 MHz, CDCl3) δ =151.85; 144.97; 142.22; 138.88; 135.21; 129.06; 121.84; 57.37; 53.74; 47.53; 35.60; 33.66; 26.64; 22.44; 19.21; 14.04; 10.15; 8.22.

HRMS (ESI, MeOH): (m/z) calculated for $[C_{25}H_{42}N_5]^+$: 412.3435; found: 412.3437

2. NMR Spectra

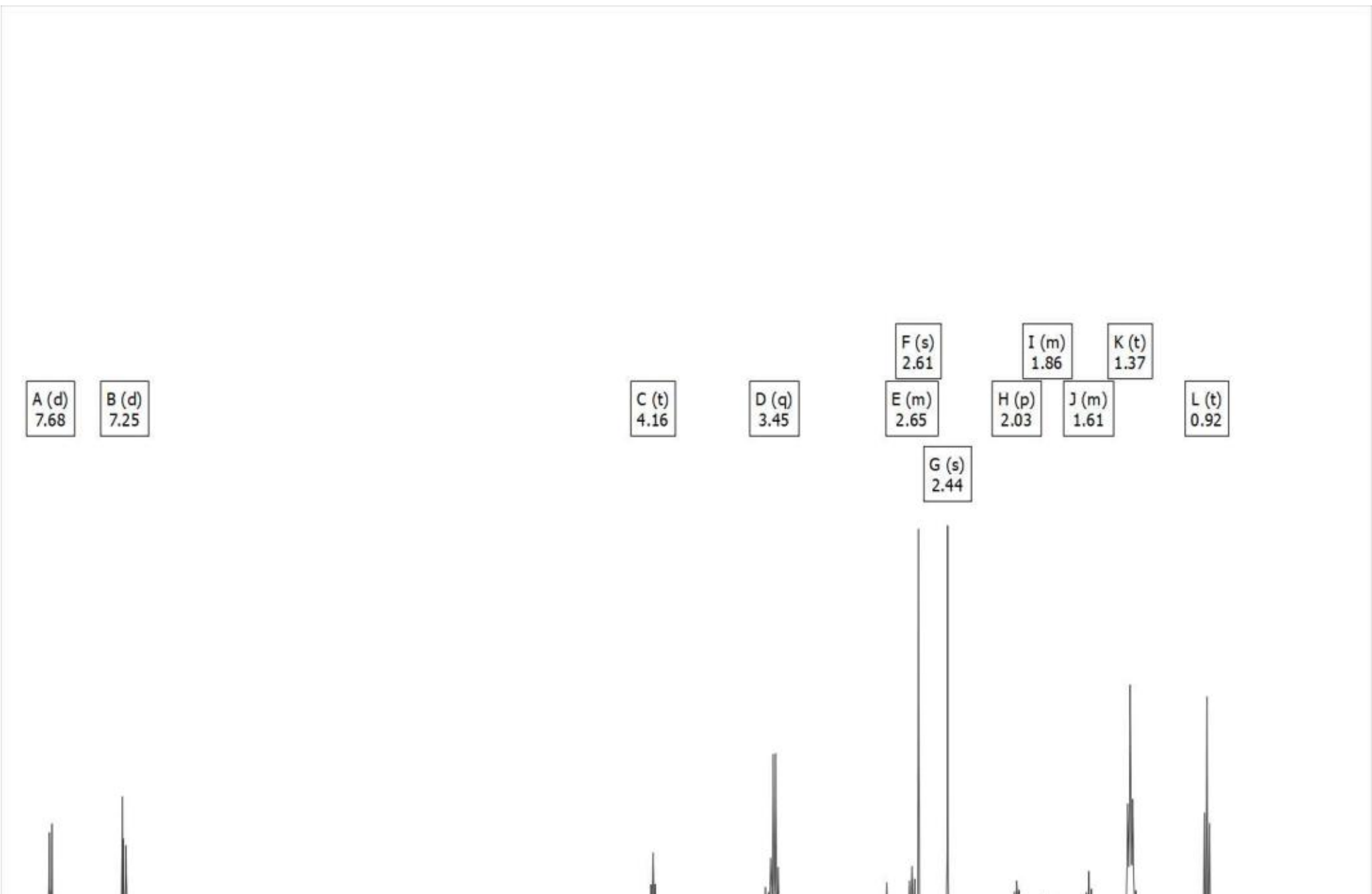

Figure S1. *^{1}H-NMR spectrum of C_4AAP-TB (500 MHz, CDCl3, 300K).*

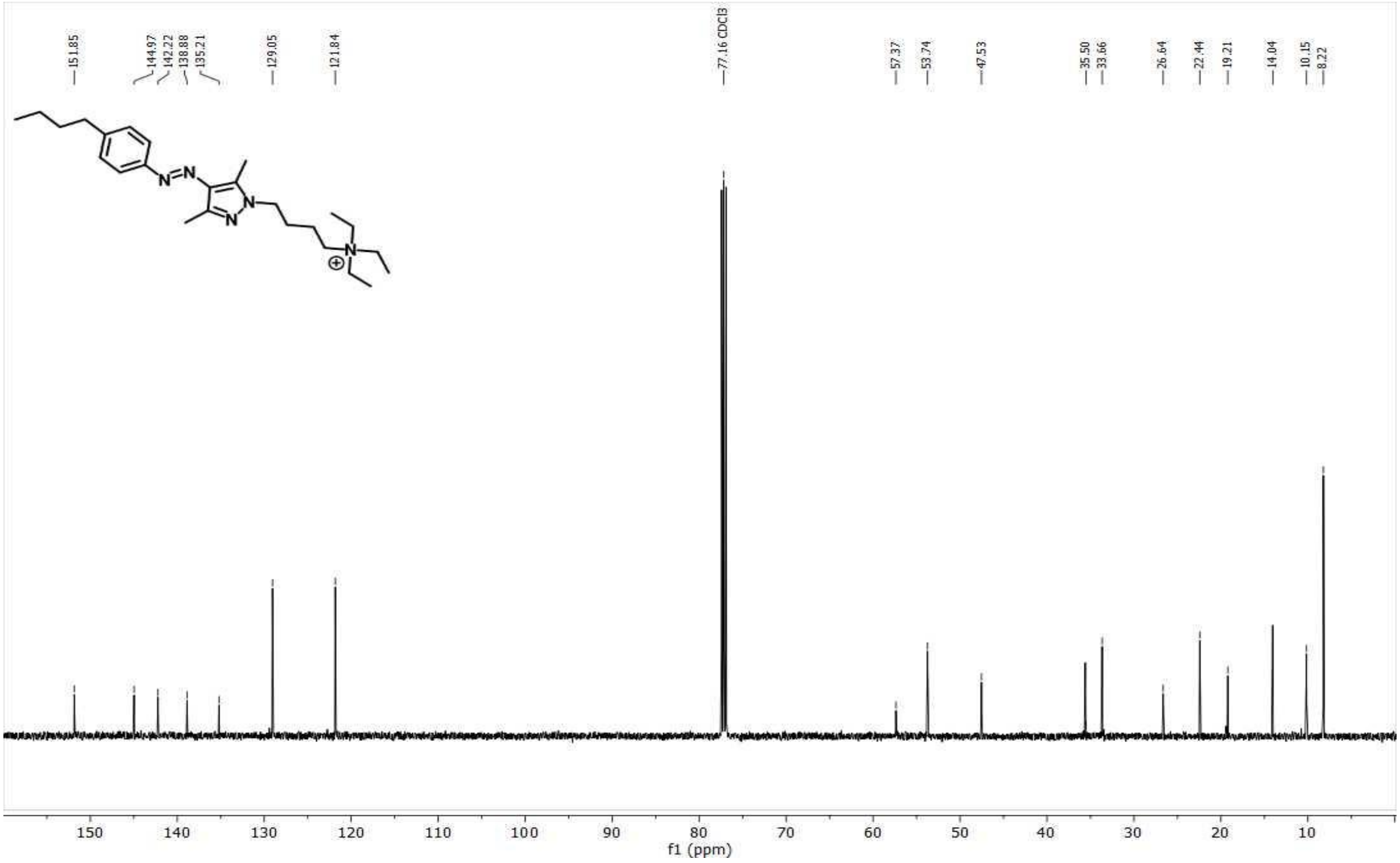

Figure S2. *$^{13}C\{H\}$-NMR spectrum of C_4AAP-TB (125.8 MHz, $CDCl_3$, 300K).*

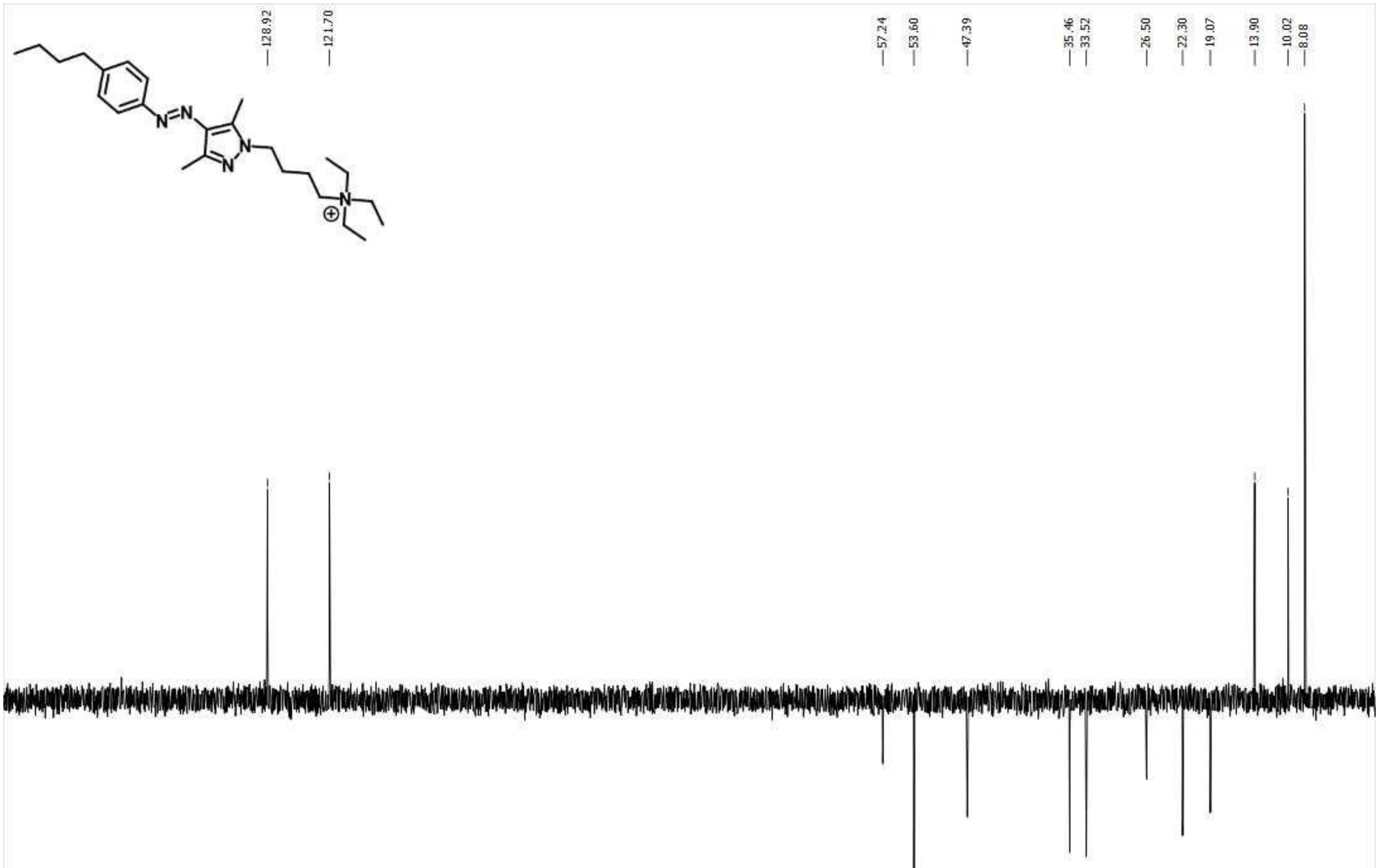

Figure S3. *$^{13}C\{H\}$DEPT135-NMR spectrum of C_4AAP-TB.*

3. Elemental analysis of Arylazopyrazole photoswitches

To determine the CHN composition of the AAPs, the powder of each AAP was weighed into tin capsules on a microbalance and combusted in an elemental analyzer (Unicube, Elementar). The gaseous combustion products were carried through the instrument and sequentially determined via thermal conductivity detector.

The element analysis for Br has been performed with an inductively coupled plasma-optical emission spectrometer (ICP-OES) (Spectro Ametek, Kleve, Germany). Gas flows were controlled by internal mass-flow controllers. A standard D-torch was employed. For sample introduction, the peristaltic pump of the system combined with a crossflow spray chamber was used under following plasma conditions: 1200 W (RF-power), 13 L/min cooling gas, 0.8 L/min nebulizer gas. Standard solutions were diluted between 10 and 100 mg/L for Bromine from single element standards (wavelength: 154.065 and 148.845 nm) with originally 1 g/L and added with 2% HNO_3 (v/v). The result of the elemental analysis is given in Table S1 below.

Table S1. *The elemental composition of arylazopyrazole photoswitches used in this study*

Compound		C (wt %)	H (wt %)	N (wt %)	Br (wt %)
$C_{21}H_{34}N_5Br$	Analysis	56.8 ± 0.3	7.8 ± 0.1	15.7 ± 0.5	31.1 ± 0.2
$C_{21}H_{34}N_5Br$	Calculated	57.79	7.85	16.05	18.31
$C_{25}H_{42}N_5Br$	Analysis	56.4 ± 0.1	8.5 ± 0.1	13 ± 0.02	25.3 ± 0.6
$C_{25}H_{42}N_5Br$	Calculated	60.96	8.46	14.00	15.99

Note that the reaction (E)-4-((4-butylphenyl)diazenyl)-3,5-dimethyl-1H-pyrazole with the butylphenyl)diazenyl)-3,5-dimethyl-1H-pyrazole (see above) to the final AAP surfactant involves actually more Br as indicated by the stoichiometry. This explains the higher values found experimentally vs the calculated ones.

4. Thermal stability of *Z* isomers for arylazopyrazole photoswitches at pH 2.2

The thermal relaxation dynamics of *Z* isomer for AAP surfactants were monitored by time-dependent UV/Vis spectroscopy (PerkinElmer Lambda 650) . For that, a solution of 0.04 mM for each AAP was prepared and then the pH value was adjusted to 2.2 using hydrochloric acid. To determine the life time of each AAP at lower pH, the prepared solution was initially irradiated with UV light (365 nm, 5 mW/cm²) until equilibrium was reached. Subsequently, the sample was kept in dark at 24 °C and absorption spectrum was recorded every one second interval about 20 min for C_0AAP-TB and 90 min for C_4AAP-TB at 335 nm center wavelength. The strong absorption band at 335 nm is assigned to the $\pi - \pi^*$ transition of the E isomer, which decreases upon $E \rightarrow Z$ photoisomerization and recovers during thermal relaxation, while the Z – isomer exhibits a weaker $\pi - \pi^*$ band. The thermal relaxation kinetics (Figure S4) were fitted with a first – order mono exponential thermal recovery model using Equation S1.

$$A_{335nm}\,(t) = A_{E-AAP} - \Delta A \cdot e^{-kt} \tag{S1}$$

where A_{E-AAP} is the absorbance of the fully relaxed E isomer at infinite time ($t \rightarrow \infty$), ΔA is the absorbance difference between the Z – rich photostationary state (A_{PSS}) and the E isomer ($A_{E-AAP} - A_{PSS}$), k is the relaxation rate constant, and τ is the lifetime of the thermal relaxation ($\tau = 1/k$). Fitting the thermal recovery yielded a lifetime of $\tau = \sim 84 \pm 0.1$ seconds for C_0AAP-TB and $\tau = \sim 10 \pm 0.1$ min for C_4AAP-TB, respectively.

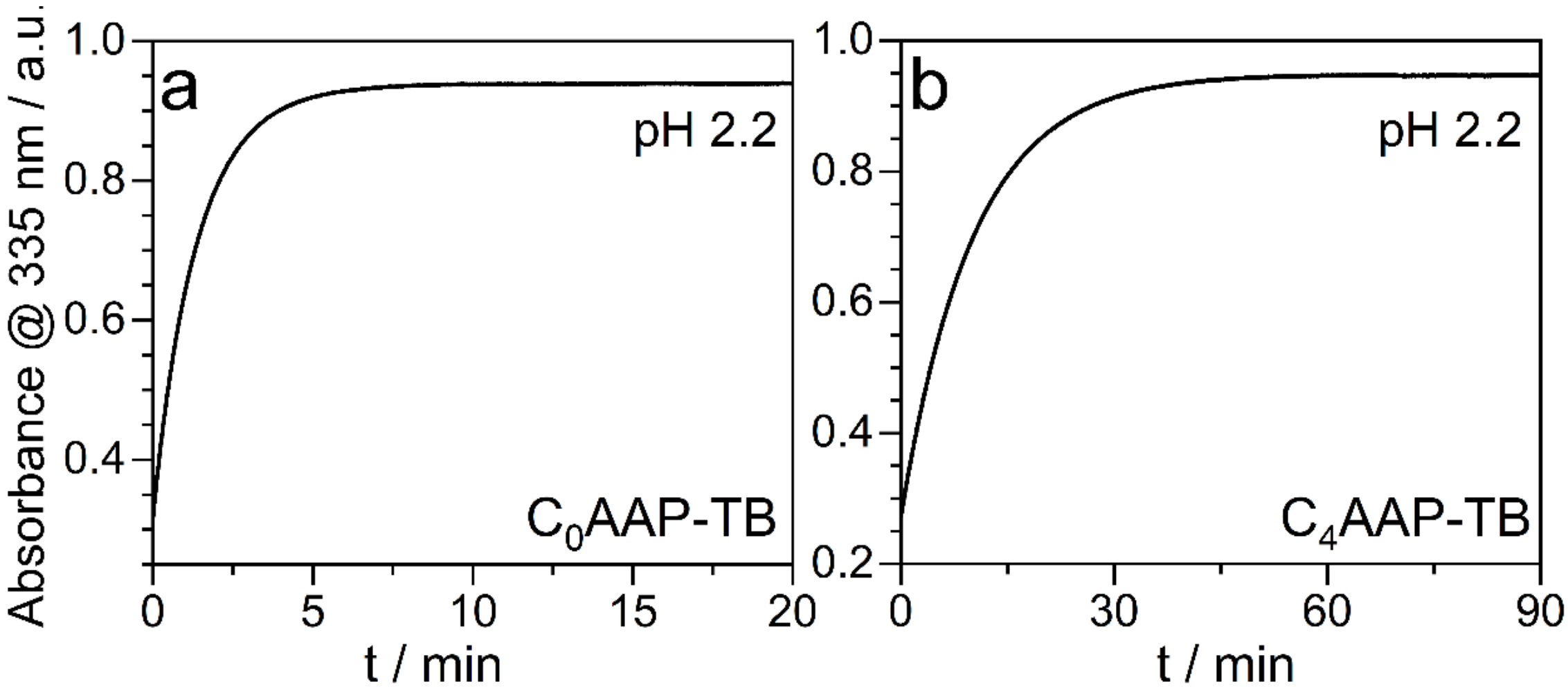

Figure S4. *Thermal relaxation of (a) Z-C_0AAP-TB and (b) Z-C_4AAP-TB at pH 2.2. After irradiation with 365 nm to the equilibrium state, the thermal relaxation of the Z isomer back to the thermodynamically stable E isomer was monitored in the dark at 24 °C using the recovery of the $\pi - \pi^*$ absorption band of the E isomer at 335 nm. Solid lines were fitted using the mono-exponential fit, which is described in the SI, yielding* τ = *~ 84 ± 0.1 seconds for (a) C_0AAP-TB and* τ = *~ 10 ± 0.1 min for (b) C_4AAP-TB, respectively.*

5. pK_a values of Arylazopyrazole photoswitches

Photometric pH titrations were carried out by recording UV/Vis absorption spectra on a PerkinElmer Lambda 650 spectrometer. To determine the pK_a of the AAP derivatives studied in this work, we followed the procedure previously described by Walther and co-workers [4]. For this determination, 0.04 mM solutions of AAPs surfactants were prepared with different pH values and irradiated with UV light (365 nm, 5 mW/cm²) for 5 min until equilibrium was reached. The corresponding isosbestic points in UV/Vis spectra (see Figure S5) at different stoichiometric ratios of the *E*- and *Z-AAP-TB* were then identified, as exemplary shown in Figures S5a and S5d. In Figure S5b and S5f, the wavelengths of the isosbestic points are plotted as a function of pH and were for further analysis first fitted with a Boltzmann function (dashed red lines), and the derivative of the latter was subsequently fitted with a Gaussian function (blue dashed lines) in order to determine determine the inflection point. The inflection point was found at pH values of ~1.6 ± 0.03 and ~1.5 ± 0.03 corresponding to the pK_a of C_0AAP-TB and C_4AAP-TB surfactants, respectively. Next, we calculated the fraction of Z-AAP that is protonated at each pH using the Henderson-Hasselbalch equation [5] (Figure S5c and S5g). In conclusion, approximately 20 % of Z-AAP is protonated at pH 2.2, which corresponds to the conditions used in the electrodewetting experiments described in the main text.

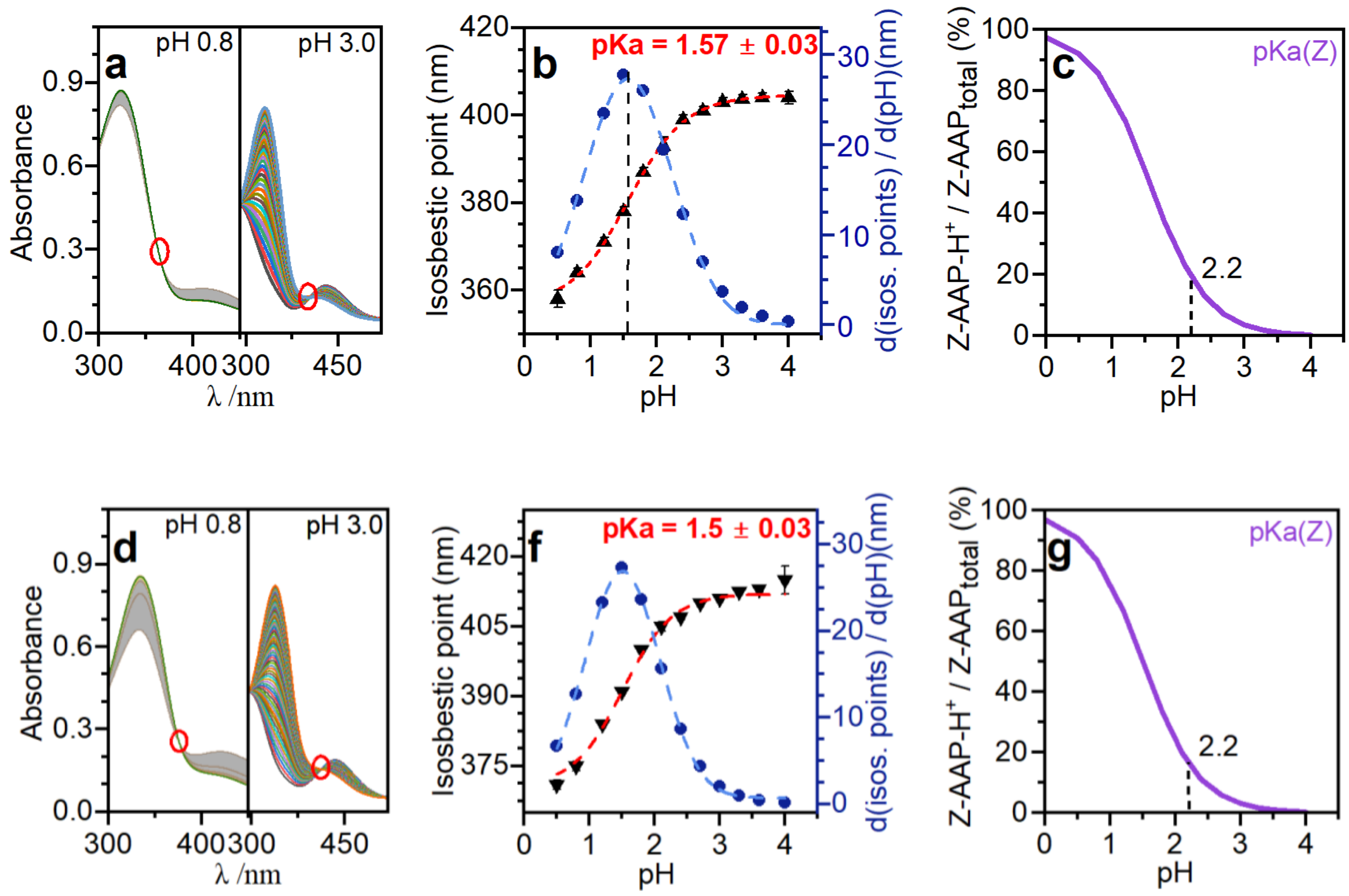

Figure S5. *Photometric determination of the pK_a values of Z-C_0AAP-TB (a,b,c) and Z-C_4AAP-TB (d,f,g). (a) and (d) present UV/Vis spectra of 0.04 mM C_0AAP-TB at pH 0.8 and 3.0, shown as examples for different stoichiometric ratios of the E and Z isomers used zo identify the isosbestic points. (b) and (f) isosbestic points (black triangles) plotted as a function of pH, from which the pK_a of Z-C_0AAP-TB-H^+ and Z-C_4AAP-TB-H^+ was obtained using a derivative (blue circles) and a Gaussian fit function. (c) and (g) show the protonation degree of Z-AAP moieties as a function of pH. The dashed lines in (b) and (f) represent fits as described in the above text of this SI.*

6. Emission spectra of the LEDs

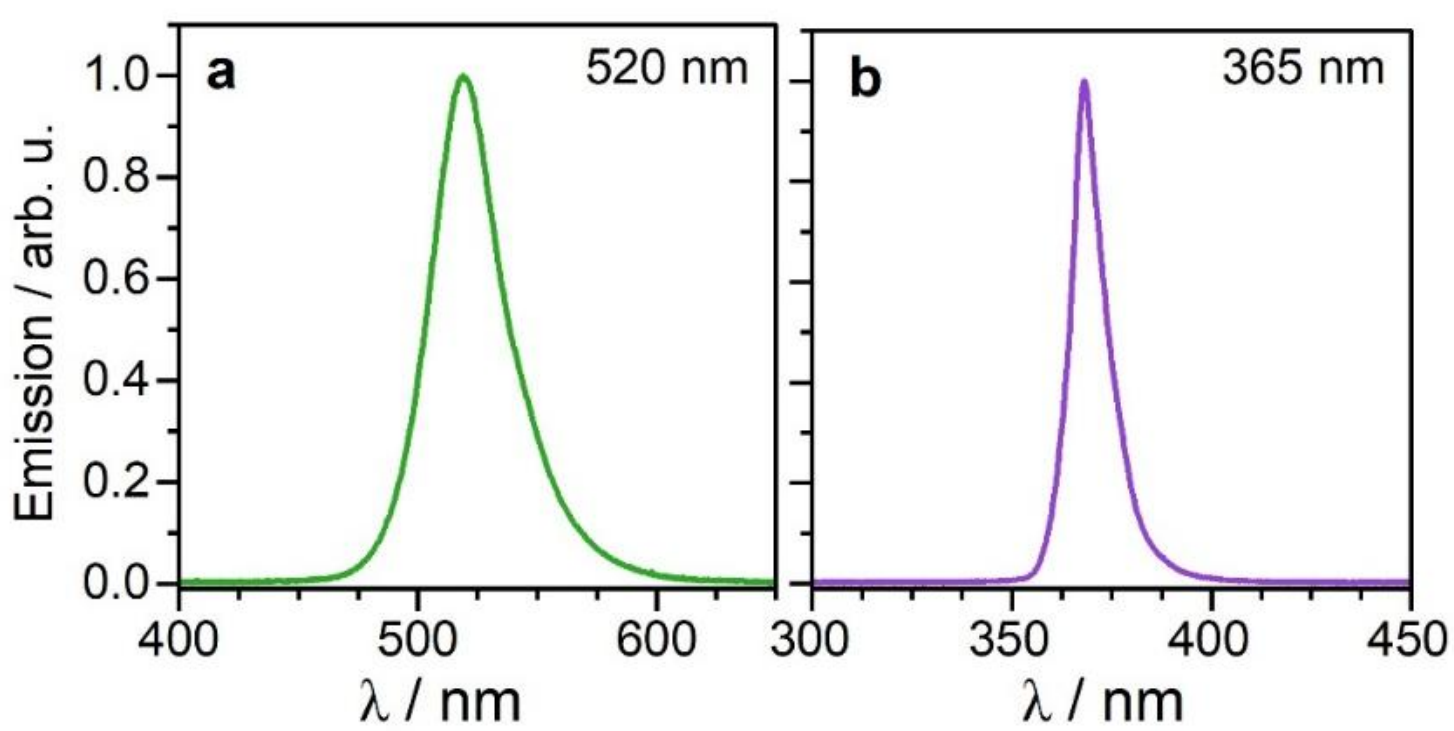

Figure S6. *Normalized emission spectra of LED used in this work (see Experimental Details in the main text) at center wavelengths of (a) 520 nm and (b) 365 nm.*

In Figure S6, the normalized emission spectra of the LEDs used in this work with emission centered at wavelength of 520 nm and 365 nm are presented. Emission was measured with a fiber-coupled UV\Vis spectrometer (Avantes; AvaSpec-ULS4096CL-EVO-UA-10).

7. UV/ Vis Spectra and Photostationary States

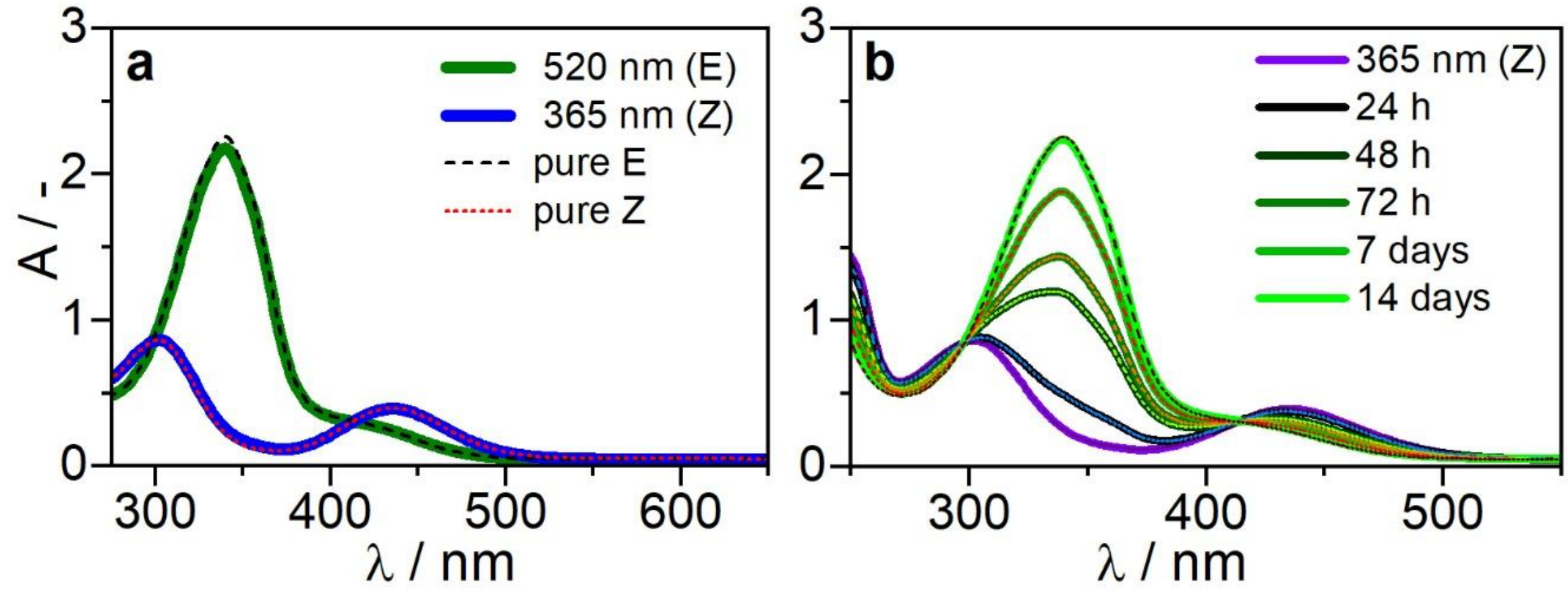

Figure S7. *UV/Vis absorbance spectra of a 0.1 mM aqueous solution of C_4AAP-TB. (a) Spectra recorded following extensive irradiation with green (520 nm) or UV (365 nm) light using LEDs with emission profiles as shown in Figure S4. The dashed red lines in (a) represent the extracted spectrum of the Z isomer from a global fit to the spectra in (b) and the pure E (100 %) spectrum that is established after equilibration in the dark for >14 days (see (b)). In (b) the Z→E thermal relaxation of C_4AAP-TB surfactants stored in the dark (without irradiation) for different times after irradiation with 365 nm light is shown. Dashed lines in (b) are fits to the spectra with different isomer ratios as explained in the text below.*

UV/Vis absorption spectra were recorded using a PerkinElmer Lambda 650 spectrometer. To obtain the data shown in Figure S7, the sample was first irradiated with green light (520 nm, 2.5 mW/cm^{-1}) for 20 minutes, subsequently a UV\Vis spectrum was taken. Next, the solution was exposed to UV light (365 nm, 5 mW/cm^{-1}) for an additional 20 minutes and UV\Vis spectrum was recorded gain.
Figure S7b demonstrates the thermal stability of the *Z* isomer with time, while the solutions are kept in the dark without irradiation for 14 days. A control sample (not shown) without any prior light irradiation was also measured after 14 days storage under light exclusion and shows a spectrum that is virtually identical with the one after UV irradiation and 14 days with light. This indicates that both samples are at or very close to 100% E isomer.
To determine the photostationary (PSS) of the E and Z isomers, we used a method previously described by Chevallier et al. [6]. For that, we assume that the total extinction coefficient of a mixture (ε_{mix}) of E and Z isomers is a linear combination of the extinction coefficient of the individual isomers (ε_{E} and ε_{Z}), weighted by their isomeric fractions f_E and f_Z with the boundary condition $f_E + f_Z = 1$. For that reason, we can write

$$\varepsilon_{\text{mix}}(\lambda)\ \ \varepsilon_{\text{E}}(\lambda)\, \text{f}_{\text{E}} + \varepsilon_{\text{Z}}(\lambda)\, \text{f}_{\text{Z}} \qquad \text{S2}$$

Using UV\Vis spectroscopy we directly measured $\varepsilon_{\text{mix}}(\lambda)$ after irradiation, while we determined $\varepsilon_{\text{E}}(\lambda)$ using the spectrum of the surfactant solution that was stored in the dark for 14 days contains about 100 % of the E isomers. $\varepsilon_{\text{Z}}(\lambda)$ was calculated by rearranging the primary equation that given above.

$$\varepsilon_Z(\lambda) = \frac{\varepsilon_{mix}(\lambda) - \varepsilon_E(\lambda)\, f_E}{f_Z} \qquad \text{S3}$$

The isomeric ratios (R_E and R_Z) under green and UV irradiation as well as $\varepsilon_Z(\lambda)$ were determined by global spectral deconvolution of the UV/Vis absorption spectra. The experimental spectra were described as a linear combination of the E and Z isomer contributions, where the E isomer spectrum

(after 14 days in the dark) was used as a fixed reference and the Z isomer spectrum was obtained by iterative nonlinear least-squares fitting. The isomeric fractions were optimized until the calculated spectra showed the best agreement with the experimentally measured spectra (Figure S7b). This was evaluated by minimization of the residuals between experimental and reconstructed absorption profiles (not shown). This approach allowed the determination of the E/Z composition of each photostationary state while simultaneously extracting the Z isomer absorption spectrum. The resulting isomer fractions obtained under green and UV irradiation for the two surfactants are summarized in Table S2 and compared.

Table S2. *Photostationary states (PSS) of photoswitchable* C_0AAP-TB and C_4AAP-TB *surfactants. Note that the values for* C_0AAP-TB *were taken from previous work [1].*

	f_E/f_Z at **520 nm**	f_E/f_Z at 365 nm
C_0AAP-TB	92 / 8	5 / 95
C_4AAP-TB	96 / 4	2 / 98
	Equilibration time after UV / days	f_E / f_Z
C_4AAP-TB	1	13 / 87
C_4AAP-TB	2	47 / 53
C_4AAP-TB	3	59 / 41
C_4AAP-TB	7	82 / 18
C_4AAP-TB	14	99 / 1

8. Determination of the Total Ionic Strengths

Table S3. *Total ionic strength of C_0AAP-TB and C_4AAP-TB solutions with different surfactant concentrations and at a pH of 2.2.*

Compound	Concentration / mM	Total ionic Strength /mM	Compound	Concentration / mM	Total ionic Strength / mM
C_0AAP-TB	0.5	6.8	C_4AAP-TB	0.04	6.35
	0.75	7.1		0.0625	6.37
	1	7.3		0.075	6.38
	1.5	7.8		0.1	6.41
	2	8.3		0.125	6.44
	2.5	8.8		0.175	6.49
	3	9.3		0.25	6.56
	4	10.3		0.35	6.66
	5	11.3		0.5	6.81
	6	12.3		-	-
	7	13.31		-	-

To determine the total ionic strength (I) we recall that for all experiments the pH of the aqueous solutions was reduced to pH 2.2 for reasons discussed in the main text. This was done by adding aliquots of concentrated HCl (35.5 %). If we assume dissociation of the strong acid at a pH of 2.2 this gives us the hydronium concentration:

$$[H_3O^+] = 10^{-pH} = 10^{-2.2} \approx 6.31 \times 10^{-3}\,M = 6.3\,mM$$

which is then the ionic strength of the solutions without added surfactants. Addition of surfactants, correspondingly adds to the ionic strength. Since, the photoswitchable surfactants used in this work are monovalent (1:1) electrolytes the overall ionic strength is a direct summation of the hydronium concentration and the surfactant concentration. The values for the ionic strength in our experiments with different surfactant concentration are shown in Table S3.

9. Electric currents and voltage dependance for electro-dewetting

As reported in the main text, small current flow across the interfaces within the drop is needed to accomplish the electodewetting of the surface. This is explained by electrophoresis of the surfactants at the air – water interface toward the three-phase contact line. We also quantified the required current for each AAP during EDeW using VersaSTAT3 potentiostat (Princeton). For that, we carried out a chronoamperometry at an applied potential of +3 V for the arylazopyrazole triethylammonium bromides (C_0AAP-TB) and +4 V for butyl-arylazopyrazole triethylammonium bromides (C_4AAP-TB) as a function of AAPs concentration. When the potential was applied, a sharp drop has been observed in current within a few seconds. To investigate the dependence of current on the concentration of AAPs solution we took the average of the current within initial two seconds after applying corresponding potential for each AAP. This was repeated for multiple independent experiments at a given concentration and the mid value of the overall of these experiments is presented in Fig. S8. From Figure S8, in general, a mean current is higher for C_0AAP-TB molecule and reaches to its maximum at about ~ 2.5 mM with a current of about ~ 4 µA, while for C_4AAP-TB molecule, the maximum occurs at 0.25 mM with a current of approximately ~ 2.5 µA. This can be attributed to the amount of charge carriers in given concentration series of the C_4AAP-TB for EDeW which is lower compared to C_0AAP-TB. Furthermore, Figure S8 shows that current flow is negligible at both low and high concentration for C_0AAP-TB (<1.5 mM and >5mM, Figure S8a) and C_4AAP-TB (<0.1 mM and >0.3 mM, Figure S8b) as it is likely there are a few charge carriers present in small concentration of AAPs, while close packed interface at high concentration decreases a migration ability of the surfactant along the water-vapor interface toward the 3-phase contact line.

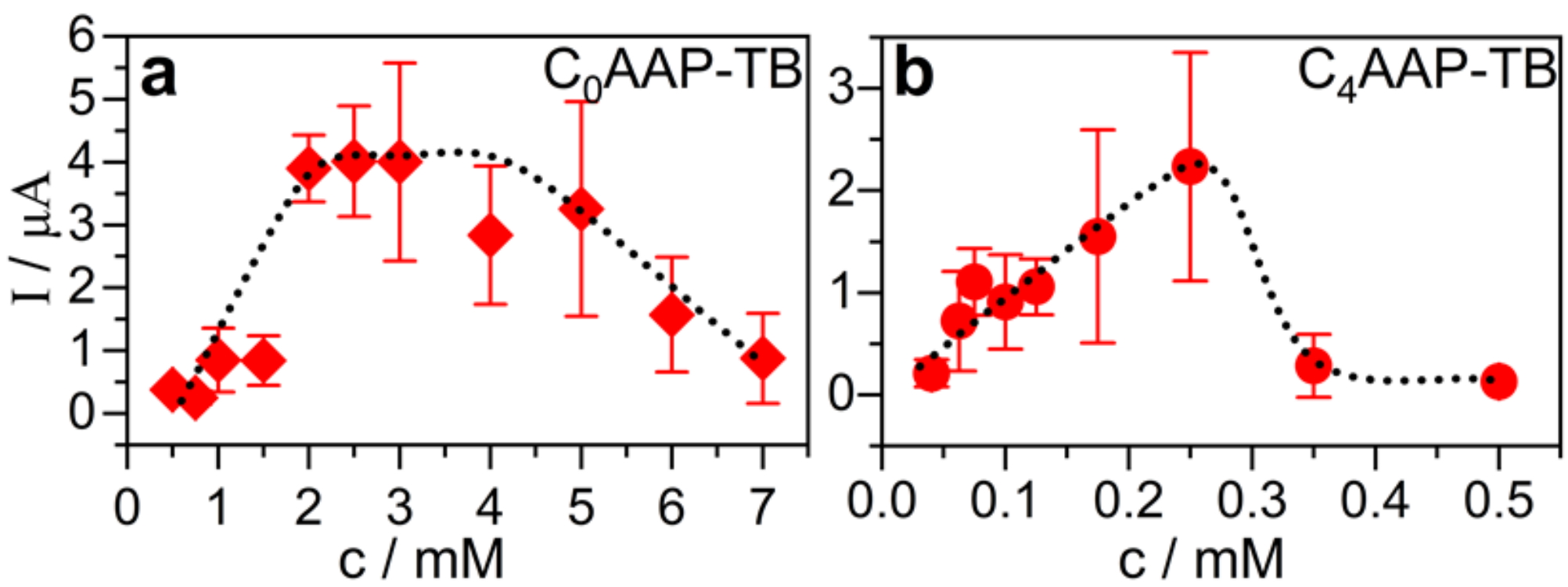

Figure S8. *Electric current I flowing between Pt wire, which employed as a working electrode, immersed in the 3 µL drop containing AAPs with a pH value of 2.2 on a Si/SiO$_2$ substrate. In the figures the mean current during the first two seconds after applying of an external potential of (a) +3V and (b) +4V for multiple repeats are shown depending on the AAP-TB concentration.*

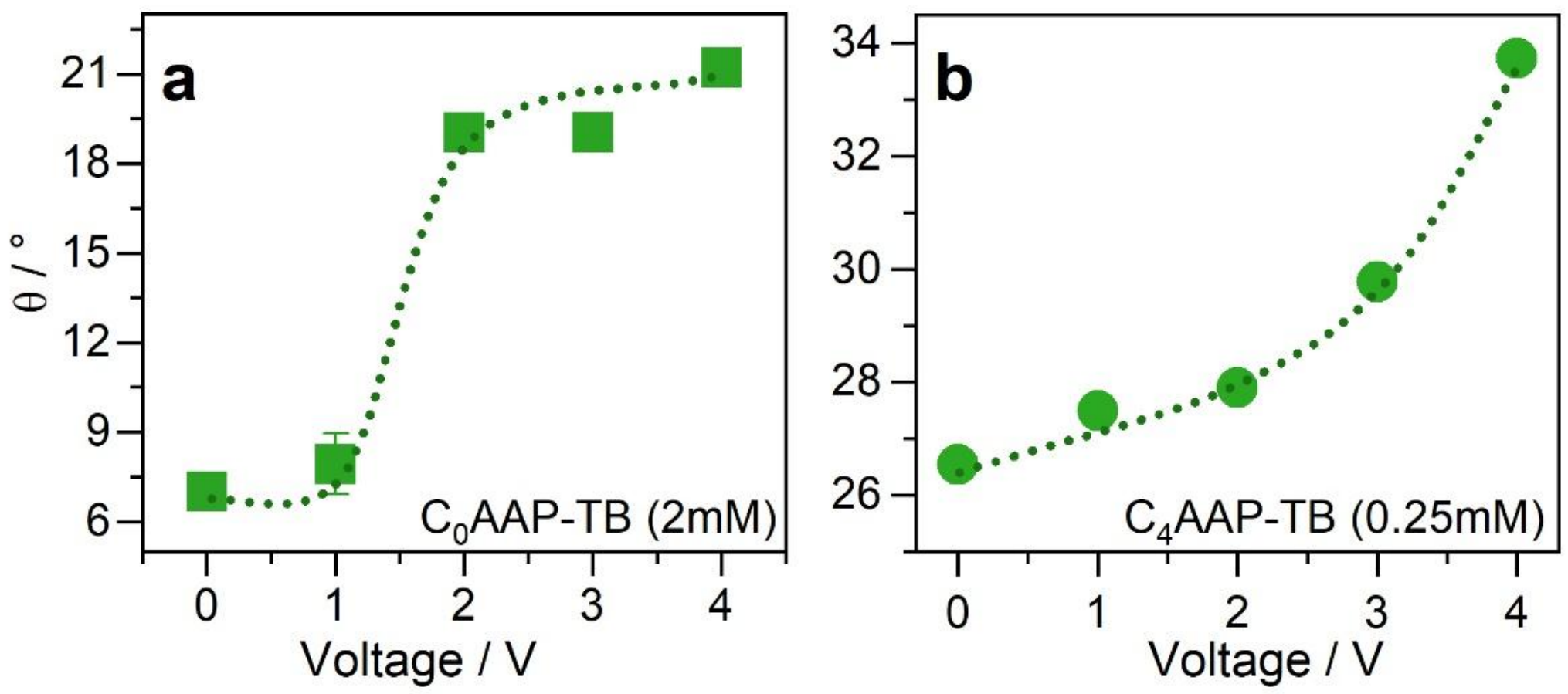

Figure S9. *Dependance of the contact angle as a function of the applied potential for (a) C_0AAP-TB and (b) C_4AAP-TB. Concentrations were as indicated in the figure and correspond to the maximum change during EDeW as shown in Figure 3 of the main text.*

References

[1] M. Schnurbus, R.A. Campbell, J. Droste, C. Honnigfort, D. Glikman, P. Gutfreund, M.R. Hansen, B. Braunschweig, Photo-Switchable Surfactants for Responsive Air–Water Interfaces: Azo versus Arylazopyrazole Amphiphiles, J. Phys. Chem. B 124 (2020) 6913–6923. https://doi.org/10.1021/acs.jpcb.0c02848.

[2] C. Honnigfort, R.A. Campbell, J. Droste, P. Gutfreund, M.R. Hansen, B.J. Ravoo, B. Braunschweig, Unexpected monolayer-to-bilayer transition of arylazopyrazole surfactants facilitates superior photo-control of fluid interfaces and colloids, Chem. Sci. 11 (2020) 2085–2092. https://doi.org/10.1039/C9SC05490A.

[3] M. Hardt, F. Busse, S. Raschke, C. Honnigfort, J. Carrascosa-Tejedor, P. Wenk, P. Gutfreund, R.A. Campbell, A. Heuer, B. Braunschweig, Photo-Responsive Control of Adsorption and Structure Formation at the Air–Water Interface with Arylazopyrazoles, Langmuir 39 (2023) 5861–5871. https://doi.org/10.1021/acs.langmuir.3c00294.

[4] S. Ludwanowski, M. Ari, K. Parison, S. Kalthoum, P. Straub, N. Pompe, S. Weber, M. Walter, A. Walther, pH Tuning of Water-Soluble Arylazopyrazole Photoswitches, Chem. – Eur. J. 26 (2020) 13203–13212. https://doi.org/10.1002/chem.202000659.

[5] H.N. Po, N.M. Senozan, The Henderson-Hasselbalch Equation: Its History and Limitations, J. Chem. Educ. 78 (2001) 1499. https://doi.org/10.1021/ed078p1499.

[6] E. Chevallier, A. Mamane, H.A. Stone, C. Tribet, F. Lequeux, C. Monteux, Pumping-out photo-surfactants from an air–water interface using light, Soft Matter 7 (2011) 7866. https://doi.org/10.1039/c1sm05378g.
